# Short-term sleep restriction in humans alters diurnal circulating metabolite profiles, including those of microbial origin

**DOI:** 10.1172/JCI189363

**Published:** 2026-03-16

**Authors:** Vanessa A. Leone, Katya Frazier, Manpreet Kaur, Evan A. Chrisler, Ashley M. Sidebottom, Ethan Tai, ViLinh Tran, Shuzhao Li, Eugene B. Chang, Dean P. Jones, Eve Van Cauter, Erin C. Hanlon

**Affiliations:** 1 Department of Animal & Dairy Sciences, University of Wisconsin-Madison, Madison, Wisconsin, USA.; 2 Department of Medicine and; 3 Committee on Molecular Metabolism & Nutrition, The University of Chicago, Chicago, Illinois, USA.; 4 Department of Medicine, Emory University, Atlanta, Georgia, USA.; 5 The Jackson Laboratory for Genomic Medicine, The Jackson Laboratory, Farmington, Connecticut, USA.

**Keywords:** Endocrinology, Metabolism, Microbiology, Behavior

## Abstract

**BACKGROUND:**

Gut microbes and their metabolites contribute to the host circulating metabolome and exhibit diurnal variation influenced by sleep-wake cycles and meal timing. Sleep deprivation alters the rhythmic circulating metabolome, but its impact on microbial metabolites remains unclear. We tested whether 24-hour circulating metabolite profiles, including those of microbial origin, differ under normal (habitual) versus short-term restricted sleep.

**METHODS:**

In a randomized crossover design, 9 healthy adults completed 2 in-lab 24-hour blood sampling sessions (q120): one following 3 nights of normal sleep (8.5 hours/night), the other following 3 nights of sleep restriction (4.5 hours/night). Meal timing and caloric intake were held constant. Serum metabolites were characterized using untargeted reverse-phase liquid chromatography–mass spectrometry and rhythmicity was assessed using empirical JTK_CYCLE analysis.

**RESULTS:**

We identified 90 metabolites, including 14 of microbial origin or derived from host metabolism of microbial products, e.g., butyrate and tryptophan derivatives. Sleep restriction significantly altered serum metabolite composition compared with normal sleep. While many compounds maintained rhythmicity across conditions, sleep restriction disrupted rhythms of several key compounds, including microbe-derived metabolites. Notably, butyrate and indole-3–propionic acid lost rhythmicity, whereas new rhythms emerged in the tryptophan catabolite, kynurenine, and lipid metabolism intermediates.

**CONCLUSION:**

We provide evidence that microbial metabolites are detectable in human blood and exhibit sleep-dependent rhythmicity. Sleep restriction alters diurnal circulating microbial and host-derived metabolite rhythms even under constant meal timing, composition, and calories. These findings support links between host sleep patterns and gut microbial metabolism and suggest microbial metabolites as potential biomarkers or mediators of sleep loss–associated health risks.

**TRIAL REGISTRATION:**

NCT00989976.

**FUNDING:**

NIH/NCRR KL2RR025000; R56DK102872-01A1, P30DK020595; P30DK042086; K01DK111785; F31DK122714; DOD W81XWH-07-2-0071.

## Abstract 2

Short-term sleep restriction in healthy human subjects alters 24-hour rhythmic profiles of circulating microbially-derived metabolites, including butyric acid and tryptophan catabolites, relative to habitual sleep.

## Graphical abstract

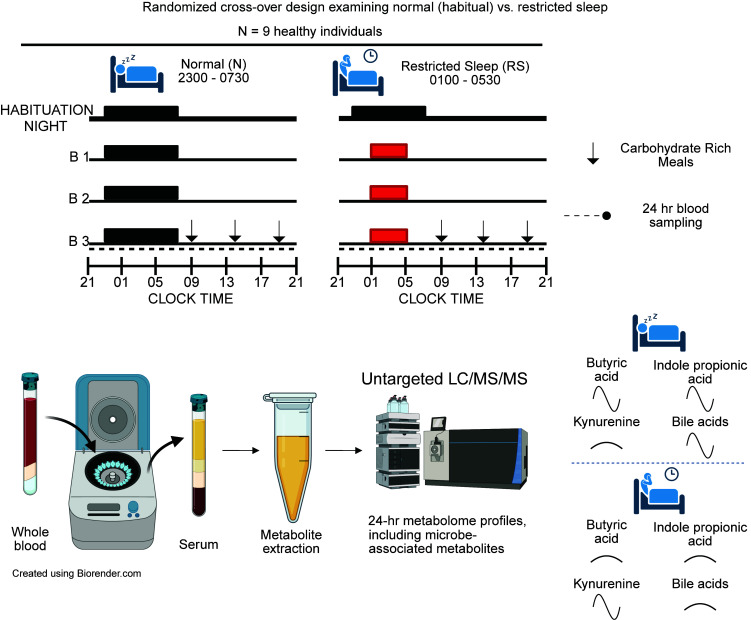

## Introduction

Biological rhythms, both internally and externally driven, are essential for maintaining health (1). Changes in our modern lifestyle, including increased exposure to light at night, mistiming of light versus dark cues, and patterns of food consumption have contributed to disruptions or shifts in biological rhythms, eliciting a profound negative impact on metabolic health (2–4). The sleep-wake cycle is a key biological rhythm conserved across invertebrates and vertebrates, which plays an important role in regulating countless processes that contribute to physiological, emotional, and mental health (5, 6). Studies have shown that sleep restriction and circadian disruption, as occur in shift work, can elicit deleterious effects on health outcomes, ranging from impaired cognition to increased risk for metabolic diseases (7–9). Despite these negative consequences, whether and how rhythmic patterns of key metabolites in circulation, including those derived from gut microbes, are influenced by changes in sleep duration remain elusive. To address this, we investigated how short-term sleep restriction alters the 24-hour (hr) rhythms of circulating metabolites, particularly metabolites of microbial origin, in human serum.

Although the methodology for examining circadian and sleep-related modulation over the 24-hr span is well established in humans, most investigations linking sleep or circadian rhythms to microbial metabolites have been done in rodents (10–13). Only a few human studies have examined diurnal variation in either host transcriptomes or circulating metabolites. For instance, in humans, abnormal sleep timing or sleep deprivation can induce changes in transcript levels, particularly in genes related to glucocorticoid signaling and immune function, suggesting they may serve as potential biomarkers of circadian phase (14). Similarly, one week of insufficient sleep altered expression of genes in blood related to circadian rhythms, chromatin remodeling, and immune/stress response (15). While these studies identified rhythms in 24-hr transcriptional profiles, both targeted and untargeted metabolomics have shown that approximately 15%–20% of detectable metabolites in plasma or saliva also exhibit diurnal rhythms (defined as 24-hr rhythmic fluctuations in levels governed by the circadian clock and/or environmental cues, including light and feeding schedules) in healthy individuals (16–20). For instance, 24-hr plasma sampling following a 24-hr wakefulness challenge showed that approximately 71% of the metabolites remained rhythmic, albeit with reduced amplitude (18). Additionally, Skene et al. showed that acute sleep deprivation alters both the amplitude and phase of numerous metabolite rhythms, indicating that sleep-wake patterns can modulate circulating metabolite rhythmicity alongside the endogenous circadian clock (20). Interestingly, these rhythmic metabolites include corticosteroids, tryptophan and derivatives, serotonin, fatty acids, amino acids, acylcarnitines, and lysophospholipids (16–18), many of which are involved in critical physiological processes, including stress response, energy metabolism, and neurotransmission.

One key group of metabolites that remain largely unexplored in this context are those of direct microbial origin or compounds produced by host secondary metabolism of microbially derived metabolites. These include short chain fatty acids (SCFAs), secondary bile acids (BAs), and aryl derivatives, which have been implicated in maintaining gut barrier integrity and regulating immune function (21), in addition to modulating host metabolic and cardiovascular health (22). Emerging evidence suggests that sleep duration and timing can influence gut microbiome composition and function, including production of these bioactive metabolites (23). For instance, in mice, we and others have previously shown that gut microbial community membership and microbial metabolites, such as the SCFA butyric acid and BAs, exhibit robust diurnal rhythms that are shaped by feeding schedules and dietary composition (10, 11, 24). In humans, circadian misalignment and timing of food intake have been shown to disrupt microbial community membership, microbial functional gene content, and 24-hr rhythms in microbial taxa, particularly in the oral microbiome (25, 26). While some human studies have also revealed rhythmicity in microbially derived metabolites, such as butyric acid (27, 28), fewer have examined how these rhythms may change in response to sleep disruption. For instance, a recent study by Bello et al. revealed that many circulating BAs, including secondary BAs produced by microbial metabolism, exhibit robust 24-hr rhythms in healthy men that were disrupted under total sleep deprivation (29). This suggests that these metabolites are sensitive to behavioral alterations, such as sleep and feeding. Given that gut microbiome disruptions are associated with conditions associated with metabolic syndrome, understanding how sleep affects the 24-hr rhythms of metabolites in circulation may provide insights into potential mechanisms linking sleep loss with risks of chronic conditions.

While informative, the majority of the studies examining the circulating metabolome have relied on total sleep deprivation, in which participants remain awake for an entire 24-hr period. However, this approach likely reflects a distinct physiological state relative to chronic partial sleep loss, a condition where individuals may obtain only 4–5 hr of sleep per night that many individuals commonly experience. Whether partial, versus total, sleep restriction similarly alters 24-hr rhythms of metabolite profiles, particularly those of microbial origin, remains unknown. Given that many of these microbial metabolites contribute to immune, metabolic, and neurological function, insights into how insufficient sleep impacts their rhythmicity in circulation could reveal novel mechanisms linking sleep and chronic disease risk. Here, we hypothesized that metabolites detectable in circulation in humans, including those that are predicted to be microbially mediated, would exhibit diurnal rhythms that are impacted by a short-term restricted sleep schedule.

**Figure 1:**
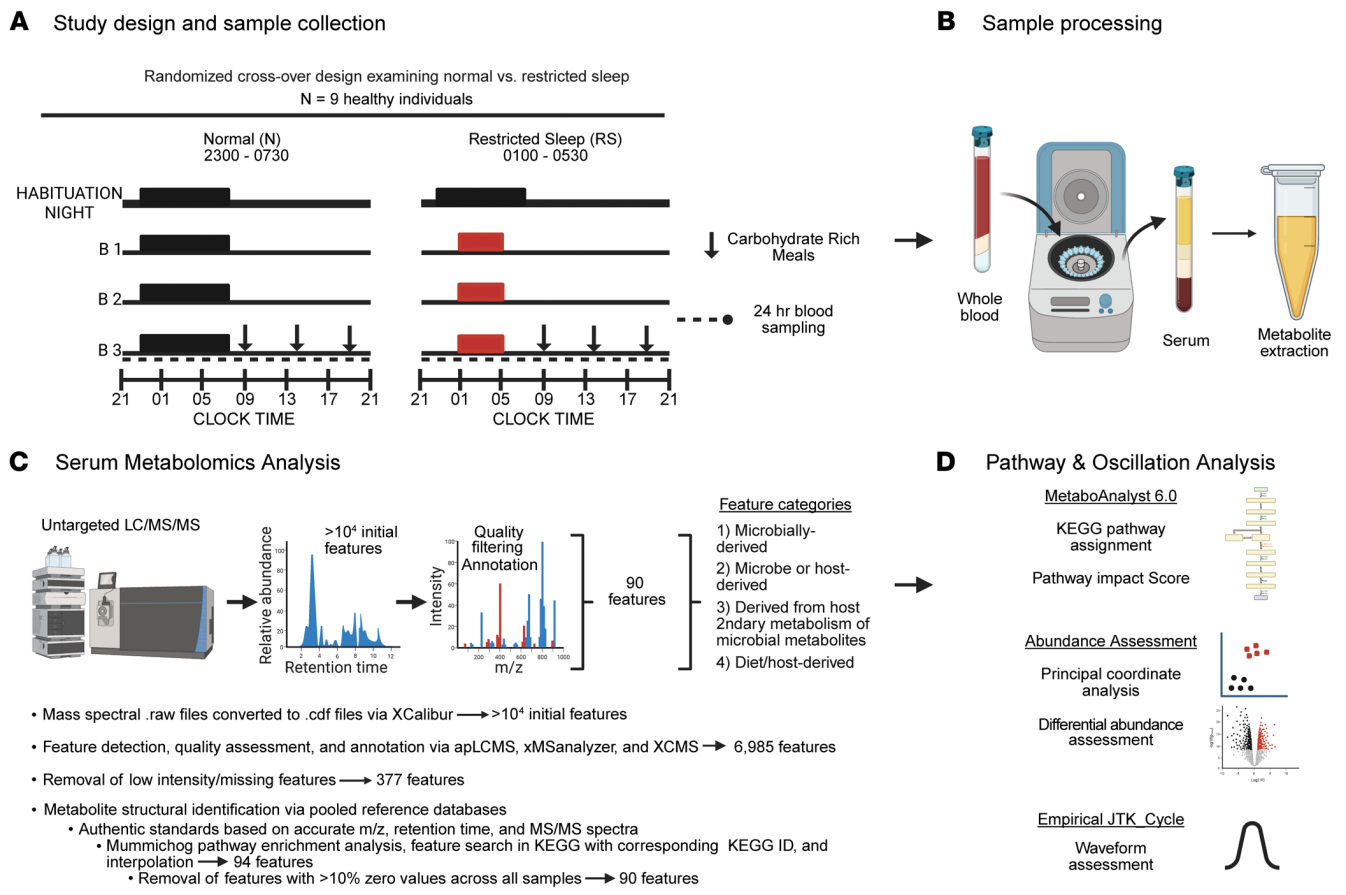
Experimental study design and workflow for serum metabolomics analysis. (**A**) Schematic of experimental design. Arrows indicate mealtimes. (**B**) Blood was collected every 2 hrs over a 24-hr period under normal sleep (8.5hrs) or short-term restricted sleep (4.5hrs) conditions and processed for untargeted metabolomics. (**C**) Untargeted LC/MS/MS workflow for processing raw data and quality control for identification of metabolite features, which were further categorized as follows: (category 1) microbially derived, reflecting metabolites exclusively derived from microbial origin, (category 2) microbe/host-derived, representing metabolites produced either by gut microbes or via host enzymatic processes, (category 3) derived from host metabolism of microbial metabolites, reflecting products of microbial metabolites that have undergone host modifications, and (category 4) diet/host-derived, reflecting metabolites absorbed from the diet and/or further processed via host metabolism. (**D**) Ninety metabolites were subsequently assigned to KEGG pathways, assessed for their 24-hr mean abundance, and examined via Empirical JTK_Cycle for rhythmic patterns. Created in BioRender. Leone, V. (2026) https://BioRender.com/zmy2qkf.

To test this hypothesis, we examined metabolites in circulation over 2 24-hr periods during normal and following acute sleep restriction. We performed 24-hr blood sampling on 9 individuals under tightly controlled in-laboratory conditions. Participant demographics are shown in Table 1, and all individuals were included in previous publications detailing daily rhythms in the endocannabinoid system under similar conditions (30–32). One female was included in our analysis based on age and BMI inclusion criteria and was not deemed an outlier in blinded metabolomic analyses. As shown in Figure 1, each participant underwent 2 separate in-lab sessions in a randomized order: one following 3 experimental days under normal (habitual) sleep conditions and one following 3 experimental days of restricted sleep (4.5-hrs/night), designed to mirror short-term sleep restriction (30–33), rather than total sleep deprivation (18, 29), constant conditions (34), or night-shift simulation (19) protocols used in previous studies. To avoid phase shifts, the midpoint of the sleep period was kept constant in both study arms by shortening sleep duration at the beginning and end of the sleep schedule during sleep restriction (Figure 1). Meal timing and caloric intake also remained fixed during both study arms.

We performed untargeted reverse-phase liquid chromatography mass spectrometry in negative mode (LC-(-)MS) to measure metabolites in serum collected from participants every 2 hrs during each 24-hr sampling period. This method allows for detection of a broad range of metabolites, including SCFAs, BAs, and phenolic compounds, that can easily gain a negative charge through loss of a proton. The objectives of this study were to (a) determine the extent to which metabolites are detected in serum collected over a 24-hr period; (b) assess whether host- or microbe-derived metabolites exhibit diurnal rhythms; and (c) evaluate whether short-term sleep restriction alters either the abundance or rhythmicity of these metabolites under conditions of fixed meal timing and composition.

## Results

### Broad categories of metabolites are detected in serum across a 24-hr period.

**Figure 2:**
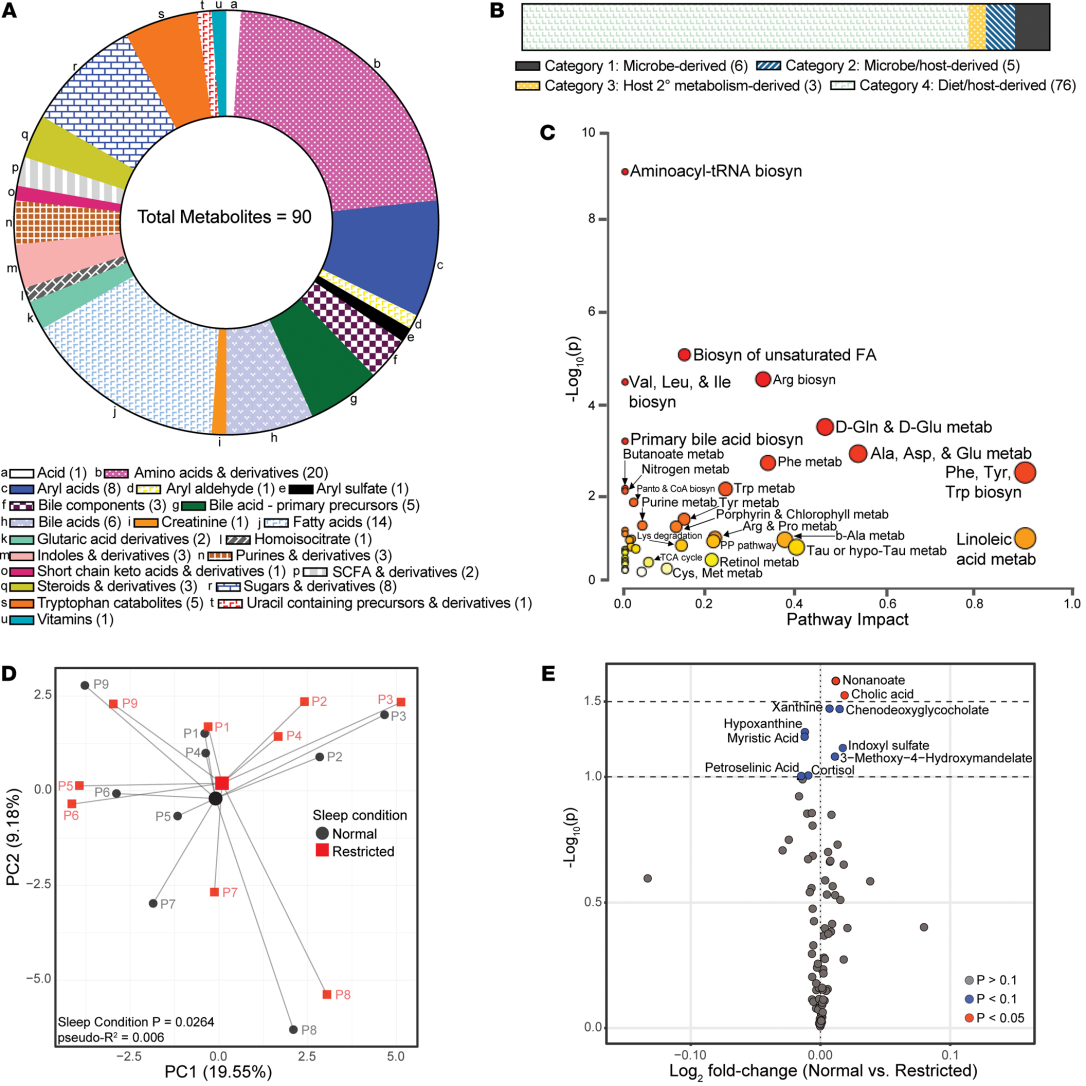
Integrated analysis and pathway enrichment reveal that short-term sleep restriction alters circulating metabolite composition. (**A**) Categorical and numerical distribution of all identified metabolites. (**B**) Categorical distribution of all identified metabolites. (**C**) Enrichment analysis (represented by –log(*P* value)) versus pathway impact (calculated from pathway topology analysis) of all identified metabolites, via MetaboAnalyst. (**D**) Principal Coordinate Analysis (PCoA) of Euclidian distances calculated from the mean relative abundance over 24-hr of metabolites under normal versus restricted sleep conditions. *P* value and pseudo-r^2^ determined using PERMANOVA implemented via the adonis2 function stratified by participant (*P* < 0.05 considered statistically significant). Participant (P) number under normal and restricted conditions indicated on graph. (**E**) Volcano plot represents differential abundance of metabolites between normal and sleep restricted conditions determined via paired student’s *t* test. The x-axis represents the log_2_ fold-change and the y-axis represents the –log_10_ *P* value. Significantly differentially abundant metabolites are highlighted in blue (*P* < 0.1) and red (*P* < 0.05).

As outlined in Figure 1, following quality assessment, annotation, and correction for retention times, 90 features were validated across all serum samples. Many of the 90 metabolites were classified as amino acids (22.2%), fatty acids (15.6%), aryl acids (8.9%), and sugars and sugar derivatives (8.9%), while approximately 16% mapped to key compounds for BA synthesis and metabolism (Figure 2A). Metabolites were further classified into one of 4 categories: (category 1) microbially derived (metabolites exclusively derived from microbial origin); (category 2) microbe/host-derived (metabolites produced either by gut microbes or via host enzymatic processes); (category 3) metabolites derived from host metabolism of microbial metabolites (products of microbial metabolites that have undergone host modifications); and (category 4) diet/host-derived metabolites absorbed from the diet and/or further processed via host metabolism. Category 4 metabolites accounted for the largest percentage of the total (84.44%), followed by Category 1 and 2 metabolites at 6.66% and 5.56%, respectively, with Category 3 metabolites accounting for the smallest percentage (3%). The number of metabolites in each category is shown in Figure 2B, and category assignments are also presented in Table 2 and Supplemental Table 1; supplemental material available online with this article; https://doi.org/10.1172/JCI189363DS1

Using the Kyoto Encyclopedia of Genes and Genomes (KEGG) database, 71 of the 90 metabolites mapped to 122 human (*Homo sapiens*; hsa) metabolic pathways, with all 71 compounds associating with at least 1 pathway (Supplemental Table 2). The microbially associated metabolites with assigned KEGG IDs mapped to 11 pathways, with 7 compounds associated with at least 1 pathway (Supplemental Table 2). Metabolites without a KEGG ID or not mapped to a hsa KEGG pathway were excluded from Supplemental Table 2. Metabolites associated with non-hsa pathways, along with their respective pathway assignments are presented separately in Supplemental Table 3.

We noted that both BAs and their precursors were detected across multiple hsa pathways, including lithocholic acid, a secondary BA produced via gut microbiota-mediated dehydroxylation of the primary BA chenodeoxycholate, which mapped to the bile secretion pathway (Supplemental Table 2). Among other microbially associated metabolites, butyric acid, a SCFA derived from microbial fermentation of complex carbohydrates, was the only compound that mapped to the hsa carbohydrate digestion and absorption pathway (Supplemental Table 2).

Several tryptophan- or indole-derived metabolites of microbial origin also mapped to general metabolism and tryptophan metabolism hsa pathways, including indole-3-acetaldehyde (IAL), indole-3-acetate (IAA), and kynurenine, the latter of which can be produced by both host and gut microbial tryptophan metabolism (Supplemental Table 2). Knurenine also mapped to the African Trypanosomiasis and the biosynthesis of cofactor pathways (Supplemental Table 2). Additionally, we detected the microbially produced indole derivative, indole-3-propionic acid (IPA), along with several metabolites derived from host secondary metabolism of indole, including indoxyl sulfate and isatin, as well as p-cresol sulfate (derived from microbially produced p-cresol); however, these metabolites did not map to any hsa pathways (Table 2 and Supplemental Table 1).

We next determined the centrality and importance of the validated metabolites within their respective KEGG hsa metabolic pathways via MetaboAnalyst 6.0 (Figure 2C). Here, a higher centrality score indicates that a metabolite occupies a critical position in connecting unique portions of a pathway, potentially influencing multiple biological processes. Similarly, a higher importance score indicates that a compound may serve as a key regulator or bottleneck within the pathway. Each identified metabolite was first mapped to the Human Metabolome Database (HMDB) and KEGG database using the MetaboAnalyst Metabolite Conversion tool, based on their common names. Verified metabolite names with KEGG IDs were then mapped to the KEGG hsa (*Homo sapiens*) pathway library. The resulting metabolite set was then analyzed using MetaboAnalyst’s targeted pathway analysis to identify associated metabolic pathways and evaluate their functional relevance.

As shown in Figure 2C, aminoacyl-tRNA biosynthesis (–log(p)=8.22, *P* = 6.35 × 10^–9^) valine, leucine, and isoleucine biosynthesis (–log(p)=3.93, *P* = 1.18 × 10^–4^), and primary BA biosynthesis (–log(p)=4.48, *P* = 3.31 × 10^–5^) were highly enriched pathways, containing 13, 9, and 4 of the detected metabolites, respectively, despite a pathway impact score of 0 in each case. This suggests that the enriched metabolites were located on the periphery of these pathways, rather than as central topological nodes. In contrast, phenylalanine, tyrosine, and tryptophan biosynthesis exhibited the highest pathway impact score (1.0) and significant statistical enrichment (–log(p)=2.09, *P* = 8.14 × 10^–3^), driven primarily by the central role of tryptophan within the pathway topology (Figure 2C).

Together, these data reveal a complex combination of host, diet, and microbially associated metabolites present in serum collected over a 24-hr period. From a pathway perspective, features related to host-mediated lipid absorption (BAs) and amino acid metabolism (tryptophan) were key contributors to the overall circulating metabolite composition.

We next performed Principal Coordinate Analysis (PCoA) to examine raw 24-hr mean relative abundances of circulating metabolites from individuals under normal and sleep-restricted conditions. As shown in Figure 2D, sleep restriction resulted in a significant shift in mean relative abundances across individuals as compared to the normal sleep condition via PERMANOVA adonis2 analysis (sleep condition *P* = 0.0264). While group-level analysis showed that sleep restriction altered overall mean relative abundances of metabolites, participant-level PCoA of paired before and after sleep-loss samples revealed statistically significant changes in 5 of the 9 individuals between conditions, indicating interindividual variability in metabolite changes following the sleep intervention (Supplemental Figure 1A). A volcano plot was generated to visualize the metabolites that exhibit differential raw 24-hr mean abundance (log_2_ fold-change) between normal and sleep-restricted conditions, which showed nonanoate, cholic acid, xanthine, chenodeoxyglycocholate, indoxyl sulfate, and 3-methyl-4-hydroxymandelate were decreased in circulation following sleep loss, whereas hypoxanthine, myristic acid, cortisol, and petroselenic acid were increased (Figure 2E). These data suggest that, despite some inter-individual variation, short-term sleep restriction shifted the 24-hr mean raw metabolite composition, with differential abundance in several metabolites driving the difference between sleep conditions.

### Consistent rhythmic patterns of serum metabolites are observed before and after sleep restriction.

**Figure 3:**
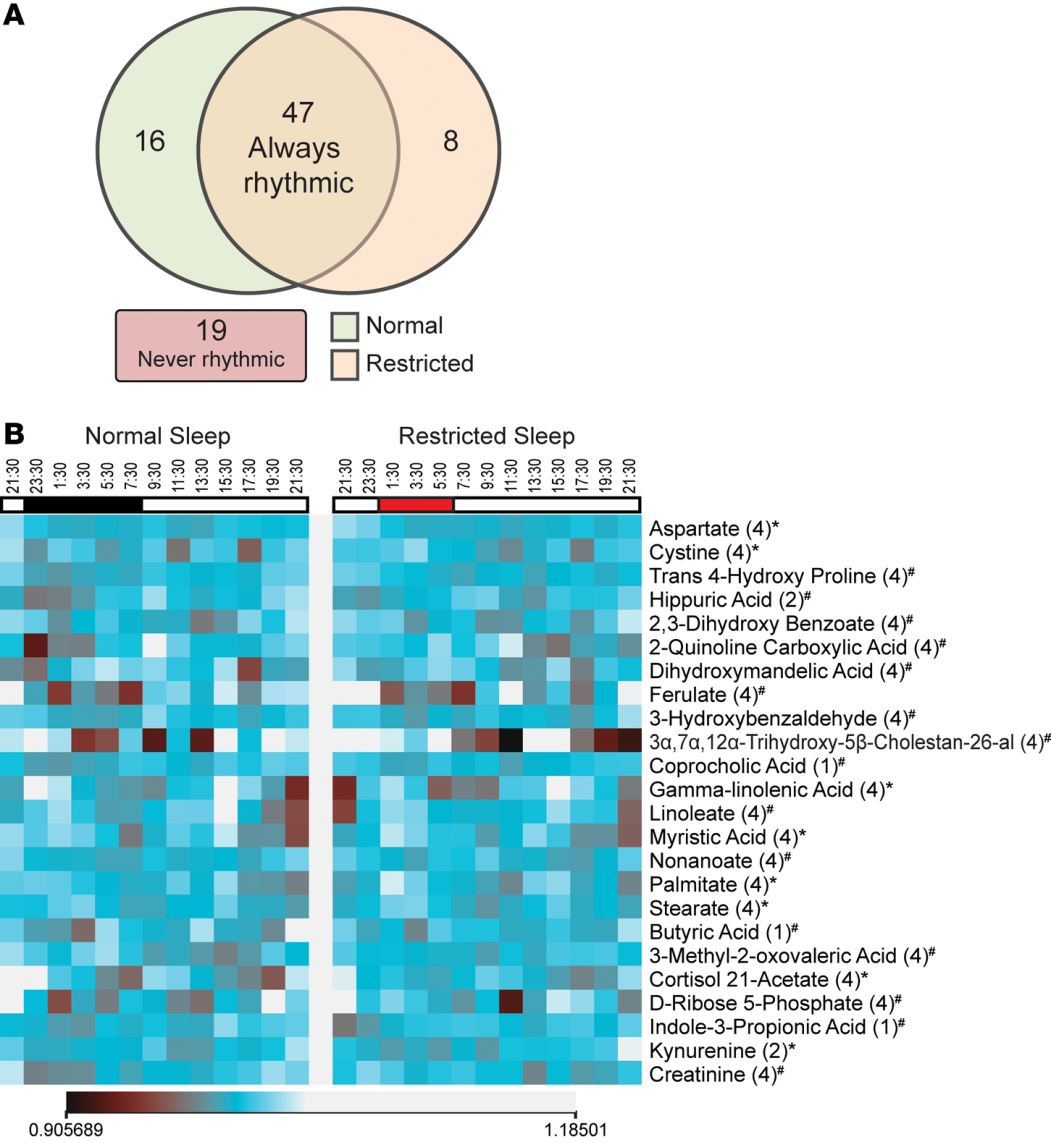
24-hr relative abundance profiles across nearly all classes of metabolites detected in circulation are differentially impacted by short-term sleep restriction, including those influenced by gut microbes. (**A**) Venn diagram depicting the number of metabolites that exhibit rhythmicity in normal sleep, restricted sleep, or both conditions determined via eJTK cycle. Box below venn diagram indicates the number of metabolites that were not rhythmic under either sleep condition. (**B**) Heatmap representing mean-normalized relative abundance of metabolites sampled every 2 hrs over 24 hrs. Black and red bars indicate sleep time in normal and restricted sleep conditions. Metabolite category is listed in parentheses and are as follows: 1, microbially derived, reflecting metabolites exclusively derived from microbial origin; 2, microbe/host-derived, representing metabolites produced either by gut microbes or via host enzymatic processes; 3, derived from host metabolism of microbial metabolites, reflecting products of microbial metabolites that have undergone host modifications; and 4, diet/host-derived, reflecting metabolites absorbed from the diet and/or further processed via host metabolism. *Indicates gain of rhythmicity following sleep restriction and ^#^ indicates loss of rhythmicity determined via eJTK cycle (GammaBH *P* < 0.05).

**Figure 4:**
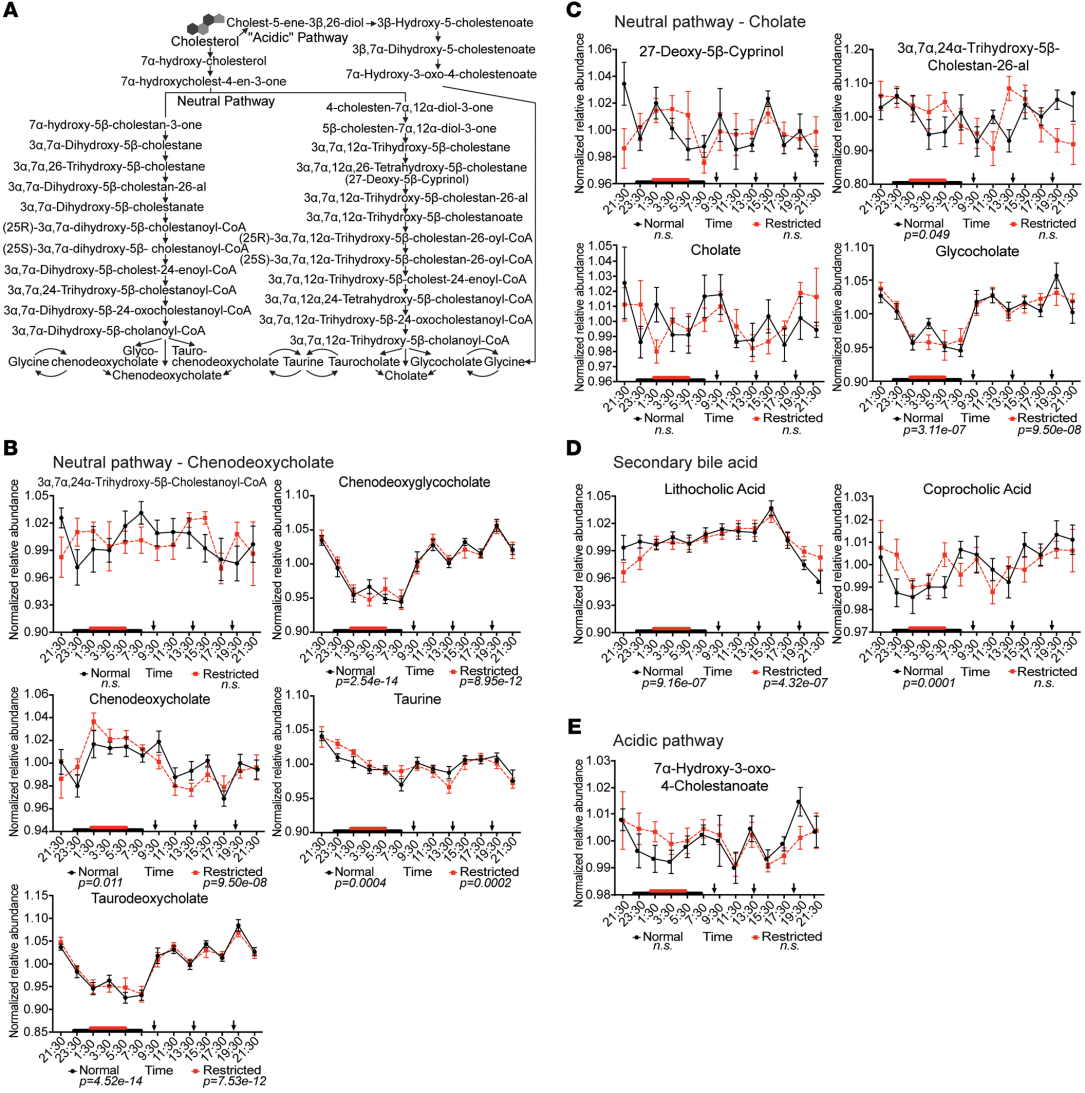
24-hr relative abundance profiles of metabolites involved in bile acid metabolism are remarkably resilient to short-term sleep restriction with fixed meal composition and timing. (**A**) Primary bile acid (BA) synthesis pathways showing precursors, intermediates, and end products, where bolded metabolites are detected in the current study. Created in BioRender. Leone, V. (2026) https://BioRender.com/2l16wwp. (**B**–**E**) Mean-normalized relative abundances of BA metabolites or their precursors detected in serum sampled every 2 hrs over 24hrs during baseline (black line) and following sleep restriction (dashed red line). Black arrows indicate meal timing. Red and black boxes on x-axis indicate sleep duration. *P* values below graphs indicate significant oscillation determined via eJTK cycle indicated. Data represent mean ± SEM.

**Figure 5:**
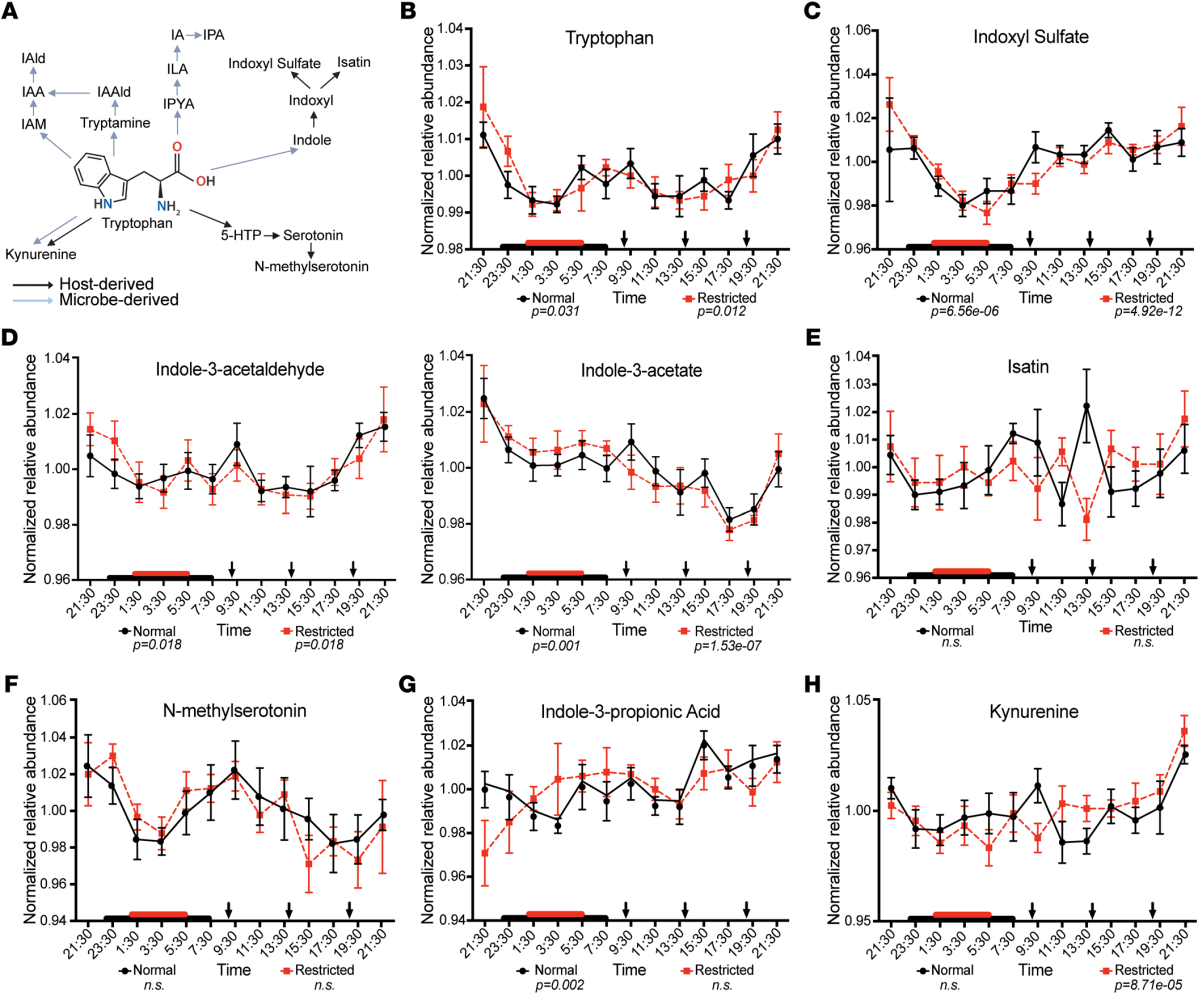
Host and microbially influenced metabolites of tryptophan metabolism exhibit unique 24-hr profiles in circulation dependent on sleep status. (**A**) Tryptophan metabolism pathways, where bolded metabolites are detected in the current study. Blue arrow indicates microbial pathways. Black arrow indicates host pathways. Created in BioRender. Leone, V. (2026) https://BioRender.com/zyldpfj. (**B**–**H**) Mean-normalized relative abundances of tryptophan and tryptophan derivatives detected in serum sampled every 2hrs over 24 hrs during normal (black line) and following sleep restriction (dashed red line). Red and black boxes on x-axis indicate sleep duration. Black arrows indicate meal timing. *P* values below graphs indicate significant rhythmicity determined via eJTK cycle indicated. Data represent means ± SEM.

**Figure 6:**
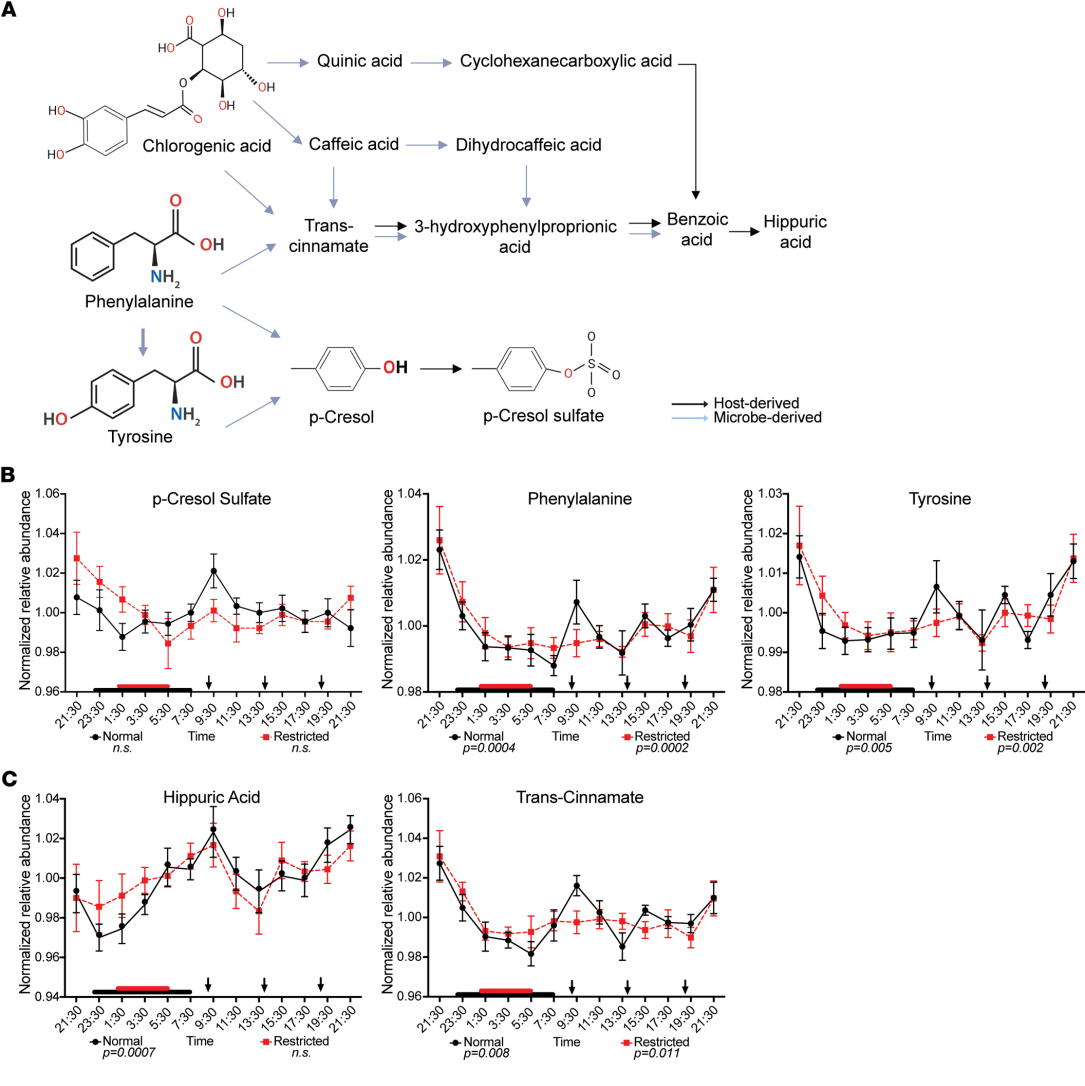
Unique 24-hr profiles of metabolites in circulation derived from host secondary metabolism of microbially derived compounds are dependent on sleep status. (**A**) Pathways depicting formation of p-cresol sulfate and hippuric acid, where bolded metabolites are detected in the current study. Blue arrows indicate microbial pathways. Black arrows indicate host pathways. Created in BioRender. Leone, V. (2026) https://BioRender.com/yytpagc. (**B** and **C**) Mean-normalized relative abundances of metabolites and their respective precursors detected in serum sampled every 2 hrs over 24 hrs during normal sleep (black line) and following sleep restriction (dashed red line). Red and black boxes on x-axis indicate sleep duration. Black arrows indicate meal timing. *P* values below graphs indicate significant rhythmicity determined via eJTK cycle indicated. Data represent means ± SEM.

**Figure 7:**
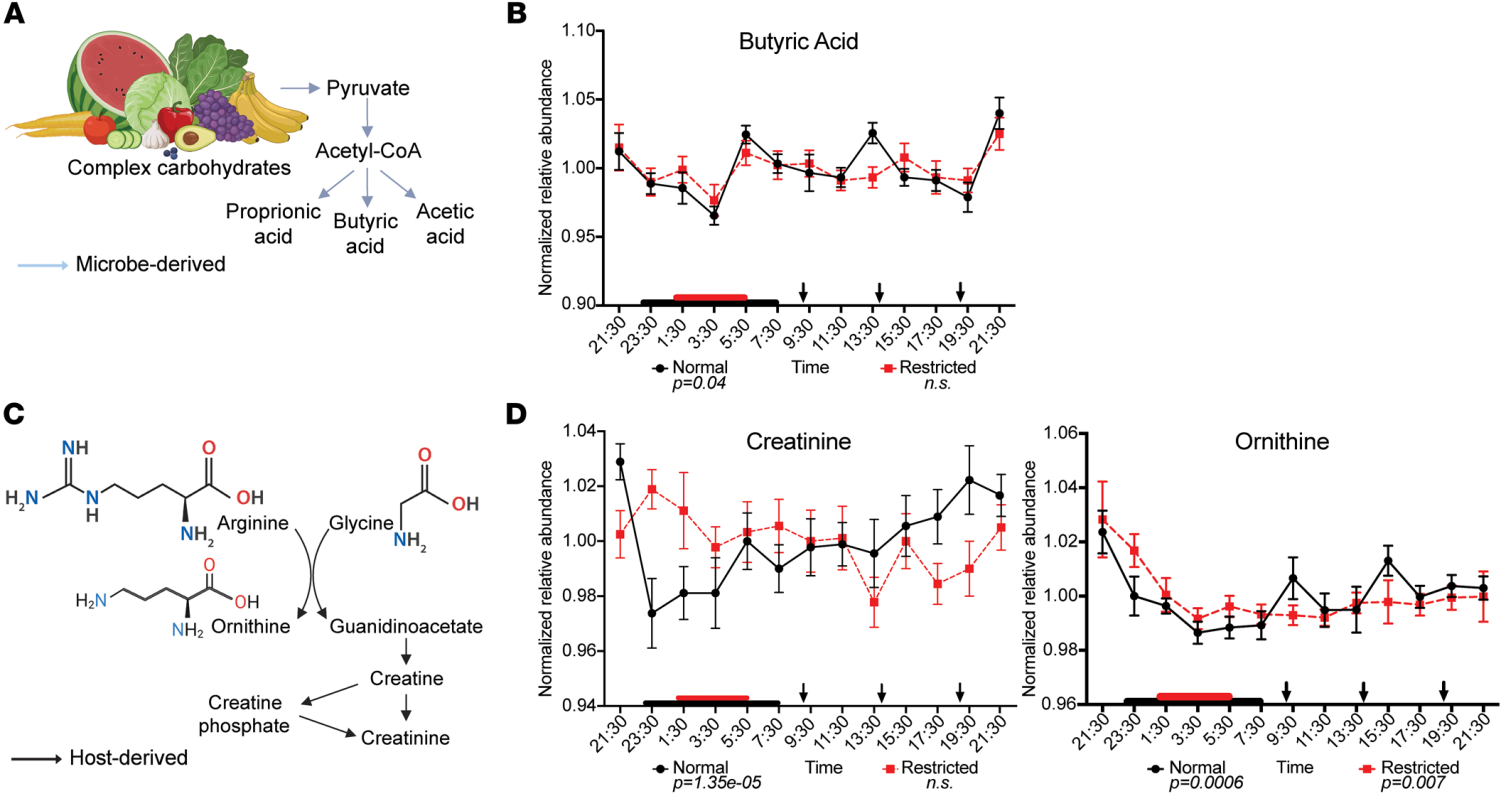
Unique 24-hr profiles of metabolites in circulation derived directly from microbial metabolism or influenced by gut microbial composition are dependent on sleep status. (**A** and **C**) Pathways depicting formation of butyric acid and creatinine, where bolded metabolites are detected in the current study. Blue arrows indicate microbial pathways. Black arrows indicate host pathways. Created in BioRender. Leone, V. (2026) https://BioRender.com/yytpagc. https://BioRender.com/b1eaa9e. (**B** and **D**) Mean-normalized relative abundances of metabolites and their respective precursors detected in serum sampled every 2 hrs over 24 hrs during normal sleep (black line) and following sleep restriction (dashed red line). Red and black boxes on x-axis indicate sleep duration. Black arrows indicate meal timing. *P* values below graphs indicate significant rhythmicity determined via eJTK cycle. Data represent means ± SEM.

To gain insight into the rhythmic patterns of the 90 metabolites under normal and restricted sleep conditions, we examined relative abundances of metabolites normalized to their means across each 24-hr collection period and applied the nonparametric empirical JTK_CYCLE (eJTK) algorithm. eJTK is a statistical method that leverages permutation-based or null distribution corrections to improve detection of rhythmic patterns, e.g., circadian or diurnal patterns (35). eJTK outcomes are presented in Figures 3, 4, 5, 6, and 7 and Supplemental Figure 1B.

We observed that approximately 52% of the 90 metabolites across all categories exhibited statistically significant rhythmic 24-hr profiles (eJTK GammaBH FDR-corrected *P* value < 0.05) during both normal and restricted sleep (Figure 3A). For instance, greater than 65% of amino acids and derivatives, greater than 57% of fatty acids, approximately 75% of sugars and derivatives, greater than 66% of steroid hormones, as well as the SCFA derivative, α-hydroxyisobutyric acid, exhibited significant rhythms regardless of sleep status (Supplemental Figure 1 and Supplemental Table 1).

Next, we considered rhythmic status based human KEGG pathway assignment to determine whether consistently rhythmic host or microbe-associated metabolites contributed uniquely to rhytmicity of functional pathways (Supplemental Table 1). As indicated by the yellow highlight in Supplemental Table 2, rhythmic metabolites were detected across numerous KEGG pathways, independent of sleep status. This included metabolites involved in primary BA synthesis and signaling, such as chenodeoxycholate, glycocholate, taurine, and bilirubin, as well as the nutritionally essential amino acid tryptophan, the latter of which is part of a broad array of KEGG pathways. 24-hr profiles of specific BAs, their precursors, and tryptophan metabolites under normal and restricted sleep are shown in Figures 4 and 5 to more precisely visualize their diurnal patterns in the context of each respective pathway.

Interestingly, all detected glyco- and tauro- conjugated BAs, the amino acid conjugate taurine, as well as the microbially derived secondary BA, lithocholic acid (LCA), exhibited robust and reproducible diurnal rhythmicity that was maintained irrespective of sleep condition (Figure 4, A–D). Furthermore, both amplitude and phase remained constant in these BAs regardless of sleep condition (Supplemental Table 4). This finding suggests that circulating BA rhythms are primarily driven by meal timing and composition (36, 37), which were consistent across both study arms, rather than by sleep status. In addition, the consistent rhythmicity observed in LCA may further indicate that gut microbial BA metabolism is more aligned with host feeding behavior, independent of sleep-wake patterns.

We next examined the rhythmicity of tryptophan and its derivatives under normal and sleep restricted conditions (pathways are outlined in Figure 5A). Previous studies have revealed the gut microbiome’s role in tryptophan metabolism and how these microbially derived metabolites connect to host health and sleep (38, 39). Here, tryptophan, indoxyl sulfate, IAL (derived from microbiota conversion of the microbial metabolite tryptamine), and IAA were significantly rhythmic under both sleep conditions (Figure 5, B–D). Similar to patterns observed in BAs, the relative abundance amplitudes and phases of most rhythmic tryptophan metabolites remained nearly identical between normal versus restricted sleep (Figure 5 and Supplemental Table 4). However, one exception was indoxyl sulfate, which remained rhythmic with similar overall abundance across conditions, but exhibited slightly elevated levels at 09:30 under normal sleep (Figure 5C and Supplemental Table 4).

In addition to tryptophan metabolites that maintained rhythmicity regardless of sleep status, we observed that hypoxanthine, a purine nucleobase produced by both host and gut microbes, known for its role in DNA replication and as an energy source for intestinal epithelial cells (40), also showed consistent rhythmicity regardless of sleep condition (Supplemental Figure 1B and Supplemental Table 1). These findings indicate that diurnal rhythms persist across a wide range of circulating metabolites, including those linked to microbial metabolism, and that such rhythms are resilient to short-term sleep restriction.

Conversely, we determined that approximately 21% of the detected metabolites were arrhythmic, regardless of sleep status (eJTK GammaBH FDR-corrected *P*-value > 0.05, Figure 3A). Notably, several BA precursors involved in primary BA synthesis and/or secretion, as well as the primary BA cholic acid, remained arrhythmic under either sleep condition (Figure 4, B, C, and E, Supplemental Figure 1B, and Supplemental Table 1). This observation may further reinforce the connection of circulating BAs to meal timing rather than sleep duration.

Both isatin and N-methylserotonin, tryptophan derivatives, were arrhythmic under both sleep conditions (Figure 5, E and F). Isatin, which is derived from host conversion of microbially derived indole, showed consistent arrhythmicity, though a qualitative difference in its relative abundance was evident at 13:30 between normal and sleep restriction (Figure 5E and Supplemental Table 4). In contrast, N-methylserotonin remained remarkably stable across all time points regardless of sleep condition, with nearly identical amplitudes and phase profiles (Figure 5F and Supplemental Table 4).

Further, p-cresol sulfate, a product of host hepatic sulfation of the microbial metabolite p-cresol in the liver (pathway shown in Figure 6A) also lacked rhythmicity (Figure 6B, left panel). However, its amino acid precursors, phenylalanine and tyrosine, retained rhythmicity in circulation under both sleep conditions (Figure 6B, middle and right panel). Like indoxyl sulfate (Figure 5C), p-cresol sulfate levels and rhythmicity were consistent between sleep conditions, with an increase observed at 09:30 under normal sleep relative to restricted conditions (Figure 6B and Supplemental Table 4).

Taken together, these data suggest that diurnal patterns in a substantial proportion of circulating metabolites are preserved following short term sleep restriction. However, even when overall rhythmicity was maintained, sleep condition still imparted modest differences in specific qualities of abundance profiles in several metabolites, including qualitative shifts in amplitude and phase (Supplemental Table 4).

### Short term sleep restriction alters the relative abundances and rhythmicity of select serum metabolites.

Despite persistent diurnal patterns in many circulating metabolites across both sleep conditions, approximately 27% of metabolites either lost or gained rhythmicity following short-term sleep restriction, with the majority (approximately 18%) losing rhythmicity relative to normal sleep (Figure 3A). Importantly, many of these metabolites were annotated to hsa KEGG pathways related to carbohydrate, lipid, or amino acid metabolism, suggesting that sleep loss may elicit subtle disruptions in temporal coordination of these core metabolic processes (Supplemental Table 2). For example, the amino acid trans-4-hydroxyproline, 3-hydroxybenzaldehyde, 2 fatty acids (linoleate and nonanoate), over half of the detected aryl acids, as well as the BA precursor 3α,7α,12α-trihydroxy-5β-cholestan-26-al, and the secondary BA coprocholic acid all lost rhythmicity under restricted sleep conditions (Figure 3B and Table 2). Interestingly, the loss of rhythmicity observed in coprocholic acid, which is derived from microbial conversion of cholic acid, contrasts with LCA, a secondary BA derived from microbial 7α-dehydroxylation of chenodeoxycholic acid, which maintained rhythmicity regardless of sleep (Figure 4D).

In addition to BAs, several other metabolites associated with the gut microbiome and known to exhibit diurnal patterns in both human and murine studies (11, 19, 28) lost rhythmicity following restricted sleep (Table 2 and Figures 5–7). For instance, IPA, a microbial derivative of tryptophan metabolism that exhibits antioxidant and antiinflammatory properties with links to improved metabolic and neurological health (41–43), lost rhythmicity following sleep restriction (Figure 5G). Further, hippuric acid (Figure 6C), derived from microbial metabolism of dietary polyphenols and associated with metabolism and kidney function (44, 45), as well as the microbial fermentation product butyric acid (Figure 7, A and B), which is known to enhance gut barrier integrity and can modulate host metabolic and immune function (46–48), also lost rhythmicity. Additionally, creatinine, which is linked to gut microbiome composition and implicated in renal dysfunction (49)*,* also exhibited this loss of rhythmicity (Table 2 and Figure 7, C and D).

We also observed qualitative differences in rhythmic abundance of these metabolites between sleep conditions. For instance, IPA and butyric acid exhibited lower relative abundance at 13:30 and 21:30, respectively, under sleep restriction (Figure 5G, Figure 7B, and Supplemental Table 4). Conversely, hippuric acid lost rhythmicity after sleep restriction (Figure 6C), likely due to elevated levels during the sleep period, while creatinine exhibited a slight phase shift in its maximum abundance compared with the normal sleep condition (Supplemental Table 4). Several precursors involved in the biosynthesis of hippuric acid, p-cresol sulfate, and creatinine — including phenylalanine, transcinnamate (a potential product of microbial metabolism), and ornithine — retained rhythmicity across both sleep conditions (Figure 6, B and C, and Figure 7D).

The microbially associated metabolites that lost rhythmicity are involved in multiple KEGG pathways. For instance, butyric acid is involved in butanoate metabolism, carbon metabolism, and protein digestion and absorption, while hippuric acid serves as an intermediate in phenylalanine metabolism (Supplemental Table 2). These findings suggest that certain classes of circulating metabolites, particularly microbial metabolites with established roles in host health, may be more susceptible to loss of rhythmicity following sleep restriction, irrespective of meal timing, and are key mediators of both carbohydrate and amino acid metabolism.

Of the approximately 27% of metabolites that exhibited a shift in rhythmic status following sleep restriction, only 8 metabolites were found to gain rhythmicity (Figure 3A and Table 2). These included the amino acid cystine, 4 fatty acids, cortisol 21-acetate (a steroid derivative), and the tryptophan derivative, kynurenine (Figure 3B). Interestingly, 4 out of these 8 metabolites are linked to lipid metabolism: the saturated fatty acids myristic, stearate, and hexadecanoic acid, as well as the ω-6 unsaturated fatty acid γ-linolenic acid. These compounds are key precursors or intermediates for fatty acid biosynthesis, degradation, and elongation (Supplemental Table 2), suggesting that short-term sleep restriction may selectively modulate rhythmicity in lipid-related metabolic pathways.

Kynurenine also gained rhythmicity primarily due to decreased relative abundance in the morning under sleep restriction compared with the normal sleep condition (Figure 5H and Supplemental Table 4). As a central metabolite in tryptophan catabolism, kynurenine has been implicated in inflammatory processes (50) and neurological disorders (51), both of which are linked to sleep disruption (52). This functional relevance is further reflected by its involvement in KEGG pathways such as African trypanosomiasis and biosynthesis of cofactors (Supplemental Table 2).

Taken together, these results reveal that sleep condition significantly alters the rhythmicity of nearly one-quarter of the metabolites we detected in host circulation, including several associated with gut microbial metabolism. Interestingly, certain tryptophan derivatives directly linked to gut microbial metabolism exhibited divergent patterns following sleep restriction: IPA, a microbial metabolite with antioxidant and antiinflammatory properties, lost rhythmicity, while kynurenine, a host- and microbe-derived metabolite linked to inflammation, gained rhythmicity. Given the distinct roles of these metabolites in host health, it is plausible that short-term sleep restriction disrupts the activity of enzymes involved in tryptophan metabolism in both host tissues and the gut microbial compartment. This possibility requires further investigation to clarify how sleep and circadian disruption modulate host-microbe metabolic interactions and their influence on host health.

## Discussion

Our findings demonstrate that nearly half of the detected circulating metabolites, spanning microbial, dietary, and host origin, exhibit robust 24-hr rhythms under tightly controlled in-laboratory conditions (Figure 3A). This rhythmicity was observed in several clinically relevant markers, such as bilirubin (liver function), creatinine (kidney function), and cortisol (linked to stress, bone, and cardiovascular health), as well as microbiome-associated metabolites, including butyric acid, IPA, and kynurenine (Table 2 and Supplemental Table 1).

Importantly, the metabolites that showed altered rhythmicity following sleep restriction include several compounds previously implicated in chronic disease risk associated with insufficient sleep, including metabolic syndrome (53–55), autoimmune disorders (56–58), multiple cancers such as breast (59, 60), pancreatic (61, 62), esophageal (63), and colorectal (64), as well as neuropsychiatric conditions, including schizophrenia (65–68). While direct evidence linking diurnal metabolite rhythms to disease incidence is still emerging, our findings demonstrate that microbially derived metabolites in circulation display measurable 24-hr patterns in humans. These observations provide a basis for investigating how circadian disruption may contribute to metabolic dysfunction and other adverse physiological outcomes, and whether such metabolites could serve as early indicators of circadian misalignment.

Previous studies have typically relied on single-timepoint sampling to examine associations between circadian patterns and disease incidence (69, 70). Our findings underscore that, for many metabolites, frequent timing of sample collection across the 24-hr cycle is a critical yet often overlooked consideration in both clinical and research protocols, which often rely on single-timepoint biomarker measurements, e.g., bilirubin, Ca^2+^, or albumin. By using a randomized cross-over design with tightly controlled sleep, meal timing, and meal composition, we isolated the specific effects of short-term sleep restriction and revealed widespread diurnal rhythmicity across multiple compound classes, including amino acids, fatty acids, aryl acids, as well as various sugars and sugar derivatives. The robustness of our untargeted LC-MS approach for diurnal detection of circulating metabolites was supported by strong correlation via nonlinear regression analysis between our LC/MS-derived cortisol levels presented here and levels derived from antibody-based detection methods (R^2^ = 0.61, data not shown).

By maintaining a fixed midpoint of the sleep cycle through restricting the start and end of sleep in a manner similar to Depner et al. (71), we minimized confounding circadian shifts that tend to complicate studies leveraging sleep deprivation via extended wakefulness in 24-hr circulating metabolites (17, 18, 29, 72). Further, our observations build upon and extend prior work by Ang et al. (16), who showed similar metabolite rhythms in 24-hr blood samples under normal sleep conditions, as well as those of Xiao et al. (73), who found associations between single-time point circulating metabolite profiles and self-reported sleep timing. Our findings contribute to a growing body of evidence that rhythmicity of circulating metabolites, particularly those linked to gut microbial metabolism, may reflect physiologically meaningful processes.

We identified that short-term sleep restriction elicits shifts in overall relative abundance and rhythmic status of a subset of circulating metabolites. For instance, metabolites that gained rhythmicity were enriched in lipid metabolism pathways, whereas those that lost rhythmicity were associated with carbohydrate metabolism (Supplemental Table 1). These shifts may suggest that changes in rhythmicity of these circulating metabolites reflect alterations in global substrate utilization in response to sleep loss. Further, microbiome-associated metabolites, in particular, were enriched in a wide range of KEGG pathways (Supplemental Table 1), indicating their potential influence on diverse host signaling processes.

While Bell et al. (72) previously showed that 8 days of restricting sleep from 8.5-hrs to 5.5-hrs elevated circulating BA intermediates, secondary BAs, and sulfated BAs at a single time point in fasted conditions, we demonstrate that both the relative abundance and 24-hr rhythmicity of many BAs and their precursors, including LCA, remained remarkably stable regardless of sleep status. Given that participants in our study were not fasted, this suggests that meal timing and composition elicit a greater influence on BA profiles than sleep duration alone (36, 37). Complementing these findings, Bello et al. (29) showed that several BAs display robust diurnal rhythms in healthy human men, and that these rhythms can be blunted by circadian disruption, highlighting the interplay between circadian regulation, meal timing, and BA metabolism. These results also align with Skene et al. (20), who proposed that the rhythms of many circulating metabolites may be governed more by peripheral clocks, such as those in the digestive tract, than by the central clock in the suprachiasmatic nucleus.

In contrast to relatively unchanged BA profiles, we show loss of rhythmicity in the microbially derived SCFA butyric acid and the tryptophan derivative IPA, both of which are associated with positive effects on immune and metabolic health, with less known about the implications for their rhythmic patterns (23, 74). Conversely, sleep restriction induced a gain of rhythmicity in kynurenine, another proinflammatory tryptophan derivative associated with depression (52) and cardiometabolic risk (75), which can be either indirectly influenced by gut microbes through modulation of tryptophan availability or via regulation of host indoleamine 2,3-dioxygenase activity (76, 77). These observations suggest that short-term sleep restriction alters diurnal patterns of host-microbe cometabolism of tryptophan. Although serotonin, another key product of tryptophan metabolism (77), was not detected in circulation in our study, these findings may indicate that other metabolites and intermediates of this pathway could serve as indicators of sleep status. Further validation in larger, diverse cohorts will be required to validate this premise. Together, our data indicate host-microbiome cometabolites are detectable in circulation and that even short-term behavioral changes, such as a few days of restricted sleep, can impact their daily fluctuations, even in healthy humans. Importantly, many of the microbial and host A subset of these metabolites could potentially serve as clinically relevant biomarkers of sleep loss.

Previous studies suggest that sleep deprivation and altered sleep schedules, e.g., shift work, can impact gut microbiota community membership (78). However, much of this work has relied on 16S rRNA gene sequencing of single, non–time-stamped stool collections, which limits insight into microbial functions and diurnal dynamics. For instance, Benedict et al. (79) found that partial sleep deprivation did not affect gut microbiota β-diversity, i.e., between subject variation, but did observe shifts in the relative abundances of specific taxa, including increases in *Coriobacteriaceae* and *Erysipelotrichaceae* and a decrease in *Tenericutes*. Despite these compositional changes, no differences were detected in fecal levels of SCFAs (79). Here, we demonstrate that the SCFA butyric acid lost rhythmicity in circulation under sleep-restricted conditions despite constant meal timing and composition.

While stool samples provide some insight into local microbial activity, there are several limitations in relying on this biomaterial alone. Stool may not reflect the extent of host absorption or downstream secondary metabolism of microbially derived metabolites in tissues such as the liver, which may be more relevant to systemic host functions. In contrast, blood offers a more integrative snapshot of host-microbe metabolic interactions and is routinely collected in clinical and research settings. Further, blood is more suitable for repeat sampling frequency over a 24-hr period, which enables investigation of dynamics that cannot be captured in stool. Future studies would benefit from combining measurements of circulating microbially derived metabolites with whole metagenomic sequencing of matched serum or stool samples to directly link specific microbial taxa with metabolite production.

Our study provides insights and proof-of-concept that broad categories of microbially influenced metabolites exhibit diurnal rhythms in human serum. However, further work is needed to elucidate the mechanisms linking sleep status to these rhythms. Although we used an untargeted metabolomics approach, metabolites were identified by matching their spectra to reference databases and metabolite libraries. Extending this approach could enable the detection of hundreds of additional known and unknown metabolites, potentially uncovering novel features affected by sleep disruption. These unbiased metabolite profiles could also support predictive modeling approaches to identify metabolite signatures associated with sleep status.

While our study was limited by sample size with inclusion of a single female, future cohorts, including more participants of both sexes would support broader generalizability. Further, the participants in our study were young, metabolically healthy adults. Future studies should examine individuals at risk for chronic diseases such as obesity and type 2 diabetes, where shifts in metabolite profiles and their rhythms may not only reflect underlying pathophysiology but also influence disease progression or severity. Although eating patterns, including both timing and diet composition, are known to influence metabolite rhythms, our study controlled for both factors, yet sleep restriction alone still altered diurnal patterns. Increasing sampling time resolution at and just after meals may help capture immediate changes in microbially derived metabolites. Whether altered dietary composition under our study protocol would elicit more pronounced shifts in the circulating metabolome similar to those observed in mice (80) remains unclear. Subsequent studies should explore how modifying diet, such as adopting a high-fat, calorie-dense diet, or altering the distribution of caloric intake across the day, e.g., early versus late meal timing, affects the rhythmicity and abundance of circulating metabolites, including those of microbial origin. Finally, extending sampling beyond a single 24-hr period would also improve both the detection and characterization of rhythmic patterns in circulating metabolites.

In summary, our findings establish that short-term sleep restriction under fixed meal timing alters the rhythmicity of circulating metabolites, including those of microbial origin. These rhythms, if validated in larger cohorts, could provide insights into the mechanistic links between sleep and metabolic health. With further work, these rhythmic host- and microbially derived circulating metabolites may form the basis for clinically relevant diagnostic and prognostic biomarkers of sleep-related health and metabolic risks, including cardiometabolic disease, inflammation, and mental health disorders.

## Methods

### Sex as a biological variable

Healthy women and men between the ages of 18 and 30, with a body mass index (BMI; in kg/m^2^) less than 28, and self-reported normal (habitual) sleep duration of 7.5–8.5-hr between 23:00 and 09:00-hrs, were recruited for participation in this study. As outlined in Table 1, only 1 female participant was included in the analysis based on age and BMI inclusion criteria and was not deemed an outlier during blinded analysis of the metabolite datasets. The female participant data was included in analysis for all metabolites except for Reichstein substance, since she did not have at least 6 data points to contribute to the 13 time points assessed over the 24-hr period (further description of metabolite inclusion criteria described in the *Statistics* section below). Given that only a single female was included, sex was not controlled for as a covariate.

### Study protocol

All study procedures took place in the University of Chicago Clinical Research Center. To exclude those with sleep disorders and diabetes, all participants underwent overnight laboratory polysomnography and a standard 75-g oral glucose tolerance test. Fasting blood sample collection was also conducted for routine laboratory analyses. Healthy individuals who did not have sleep disorders and had normal glucose tolerance were included. Exclusion criteria included: irregular sleep schedule, habitual daytime naps, shift work, travel across time zones in last 4 weeks, chronic medical condition, acute illness, use of any prescription medications, use of over-the-counter medications or supplements known to affect sleep or glucose metabolism, smoking, marijuana usage, excessive alcohol (> 2 drinks per day) or caffeine (> 300 mg per day) consumption, history of psychiatric disorders, or abnormal findings on medical history, physical examination or routine laboratory testing; further exclusion criteria have been detailed in a previous publication (31).

As described previously (31), each participant was tested under 2 sleep conditions, in randomized order, spaced by at least 4 weeks (Figure 1A). Participants were instructed to maintain a standardized schedule of bedtimes (23:00 – 07:30) and not to deviate from this schedule by more than 30 minutes during the week preceding each sleep condition. In the week prior to each in-lab sleep condition, sleep-wake cycles of the participants were continuously monitored by wrist activity (Actiwatch; Philips Respironics, Bend, OR) to verify adherence to instructed standardized schedule of bedtimes (23:00 – 07:30). The 2 in-lab sleep conditions consisted of a session of normal sleep condition; 3 consecutive in-laboratory days with 8.5-hr in bed (23:00 to 07:30, normal sleep), and restricted sleep session; 3 consecutive in-laboratory days with 4.5-hr in bed (01:00 to 05:30, restricted sleep, RS). Each condition was preceded by 1 night of acclimation to the laboratory environment with 8.5-hr in bed. No naps were allowed during either session.

During in-lab sessions, polysomnography was conducted each night, with sleep characteristics detailed in a previous publication clearly showing the successful implementation of the sleep restriction (31). In brief, during the restricted sleep condition, participants slept on average 251 min/night, whereas during the normal sleep condition they slept on average 453 min/night (*P* < 0.0001). The reduction in total sleep time was due to decreased time in Stage N1 and N2, as well as a reduction of the time spent in REM sleep. Time spent in deep NREM sleep (Stage N3) was nearly identical during restricted as compared with normal sleep. In both the normal and restricted sleep conditions, the lighting schedule was such that only dim light (ceiling lights off, shades closed) was allowed in the participant’s room from wake until 10:30 and again from 18:00 until bedtime, irrespective of sleep/wake time. Ceiling lights and open shades were approximately 550–600 lux, whereas dim light was approximately 75–100 lux (EXTECH Light Meter). Participants were housed in a private room and limited to sedentary activities during waking hours. Compliance to all aspects of the protocol was monitored continuously by research staff.

In the afternoon, following the second night of each condition, an intravenous sterile heparin-lock catheter was inserted in a forearm vein. The line was kept patent with a slow drip of heparinized saline. Blood sampling was initiated prior to bedtime on the third night in each condition (B3/R3) at 21:30 at regular intervals and continued for 24-hrs over the fourth experimental day (Figure 1). Microbiome metabolites were measured at 120-min intervals. During bedtimes, the catheter was connected to plastic tubing that extended to an adjacent room to sample distally without disturbing the participant. Samples were collected at room temperature in tubes that did not contain inhibitors. Serum samples were frozen at –80°C until analysis.

### Controlled caloric intake

A registered dietitian from the Clinical Resource Center Metabolic Kitchen supervised the preparation of all meals. Caloric intake was identical under both sleep conditions and was strictly controlled. Caloric content of meals was calculated by a bionutritionist to meet individual participant’s caloric requirements for sedentary conditions (81). Participants were not allowed to consume any foods or beverages that were not provided by the metabolic kitchen. For the in-laboratory days prior to 24-hr sampling, standardized meals were provided to participants with macronutrient composition of 55%–60% carbohydrate, 15%–20% protein, and 30%–35% fat. During the 24-hr period of blood sampling, participants ate 3 identical carbohydrate-rich meals (20% fat, 68% carbohydrate, 12% protein). All meals were served at 09:00, 14:00, and 19:00 and participants were instructed to consume each meal in its entirety within 20 minutes. Water was freely available and could be consumed ad libitum.

### Mass spectrometry

#### Sample preparation and feature detection.

Serum samples were analyzed using a broad, untargeted LC-MS/MS platform. The samples were block randomized, so that longitudinal samples of a participant were included and randomized in a batch, and individual participants were randomized across batches. Samples were prepared and analyzed along with pooled reference human serum as previously described (82). The same NIST SRM 1950 material and Qstd3 samples used in this publication were analyzed along with the current study samples. Briefly, 50 μL of sample extracted with 100 μL of acetonitrile containing a mixture of 9 stable isotope internal standards, centrifuged to pellet proteins and supernatants were transferred to a 4°C autosampler for analysis. Aliquots of 10 μL were analyzed with three technical replicates using hydrophilic interaction liquid chromatography (HILIC) with negative electrospray ionization and Fourier Transform high-resolution mass spectrometry (Dionex Ultimate 3000, HF Q-Exactive, Thermo Scientific). Separation of the extract was performed on a Waters XBridge BEH Amide XP HILIC column (2.1 mm x 50 mm, 2.6 μm particle size) and gradient elution with mobile phases A: LCMS grade water, B: LCMS grade acetonitrile, C: 2% formic acid. The initial 1.5 min period consisted of 22.5% A, 75% B, and 2.5% C, followed by a linear increase to 75% A.

#### Data analysis.

Mass spectral files in.raw format were converted to.cdf files using XCalibur file converter software (Thermo Fisher, Waltham, MA) and extracted using apLCMS (83) and xMSanalyzer (84) to generate feature tables which contain mass spectral features defined by mass-to-charge ratio (m/z), retention time, and ion abundance. Low intensity and/or missing features were removed if the feature was not observed in two or more replicates. Retention times were corrected across all samples with XCMS (xcmsonline.scripps.edu). Initial metabolite values represent relative abundances normalized to internal standards.

Metabolite detection and processing were conducted in an unbiased, untargeted fashion. While we anticipated detection of a subset of known microbially derived or microbially-influenced metabolites would be detected based on our prior work (11) and from others (28, 29, 85), no compounds of microbial origin were pre-selected or filtered. Microbiome-associated metabolites were identified *post hoc*, during annotation and downstream interpretations, using libraries, existing literature, and publicly available databases.

Metabolite identification was performed at multiple levels and is outlined for each metabolite in Supplemental Table 5. Level 1 identification included metabolites with accurate mass match (< 5 ppm) and coelution within ± 0.5 min of confirmed metabolites in the pooled reference serum (86), validated against authentic standards using accurate mass *m/z*, retention time, and MS/MS spectra (82). Features from *mummichog* pathway enrichment analysis (87) with accurate mass match (<5 ppm) to common adducts [M-H]^–^, [M-2H]^2–^, [M-H_2_O-H]^–^ were considered Level 3 identification (86) based upon accurate mass match and pathway association. Remaining features were searched in the Kyoto Encyclopedia of Genes and Genomes (KEGG) with the corresponding KEGG ID and considered Level 5 identification (86). Each identified metabolite was then mapped to the Human Metabolome Database (HMDB) and KEGG databases through their common name using the Metaboanalyst Metabolite Conversion tool. The HMDB and KEGG IDs were manually validated, and compounds that did not have a KEGG ID were not included in downstream analysis. The reference library utilized for annotation includes a wide range of metabolites of endogenous, dietary, pharmaceutical, and microbial origin.

Following identification and annotation, each metabolite was assigned to one of four categories based on origin. Category 1 (microbially derived) reflects metabolites exclusively derived from microbial origin, e.g., SCFAs. Category 2 (microbe/host-derived) represents metabolites produced either by gut microbes or host enzymatic processes, e.g., kynurenine. Category 3 (host metabolism of microbially derived metabolite) represents products of microbial metabolites that have undergone host modifications, e.g., indoxyl sulfate. Finally, Category 4 (diet/host-derived) reflects metabolites absorbed from the diet and further processed via host metabolism, e.g., hippuric acid derived from dietary polyphenols.

Pathway analysis was performed using the Pathway Analysis module in MetaboAnalyst 6.0 with the *Homo sapiens* KEGG pathway library as the reference. Identified metabolites were first mapped to HMDB and KEGG identifiers using the MetaboAnalyst Metabolite Conversion tool based on common names. Metabolites with verified KEGG IDs were subsequently subjected to targeted pathway analysis against the KEGG hsa pathway library to identify enriched metabolic pathways and assess their functional relevance.

All MetaboAnalyst analyses were performed using default parameters. Data normalization and transformation (sum normalization, log transformation, and autoscaling) were applied automatically. A Hypergeometric test was used for pathway enrichment, and relative betweenness centrality was used for pathway topology analysis to evaluate metabolite importance based on node connectivity, pathway position, and effect scores. The entire Homo sapiens metabolome (from KEGG) served as the background set for enrichment calculations.

MetaboAnalyst generated a scatterplot to visualize pathway analysis results, which displays matched pathways ranked by p-values (enrichment) and impact scores (topology). Metabolite nodes were color-coded from yellow to red, indicating increasing levels of statistical significance within each pathway.

To visualize overall metabolomic composition by participant and sleep condition, irrespective of time, a participant-level raw relative abundance matrix was assembled. Average peak intensity was calculated over time for each metabolite and each participant, and the resulting values were then log_10_ transformed. Euclidean distances between participants under normal versus restricted sleep conditions were calculated, and Principal Coordinate Analysis (PCoA) was performed using functions from the vegan package (v2.7-2) in R (88).

### Statistics

Participants were excluded from analysis for individual metabolites if they did not have at least 6 data points to contribute to the 13 time points assessed over the 24-hr period. Considering this criterion, only 8 participants contributed to the analysis of 3 metabolites: D-ribose R-phosphate, Reichstein’s substance S, and IPA. Missing and isolated values that represented a relative change of more than 100% in comparison with both the preceding and following values were assumed to represent assay error and were replaced using linear interpolation. During the normal sleep condition, of the 11,213 data points, 66 time points (or 0.58%) were interpolated. During the restricted sleep condition, of the 11,104 data points, 68 time points (or 0.61%) were interpolated. Four metabolites (taurolithocholate, n-formylglycine, ethanolamine phosphate, and chenodeoxycholate) exhibited greater than 10% 0 values across all measured timepoints and hence were excluded from further analyses. The raw abundance values for the 90 resulting metabolites were normalized to the mean 24-hr abundance, centering the data on 1. 

To test whether global metabolite composition for the raw abundance values for the 90 resulting metabolites differed by sleep condition, PERMANOVA was performed using the adonis2 function in vegan (v2.7-1) in R (88), which is based on the previously described method by Anderson (2001) (89). Permutations were constrained within participant ID, so that condition labels were only permuted within participant, controlling for interindividual baseline differences. Statistical comparisons between sleep condition are reported as PERMANOVA pseudo-R^2^ (variance explained) and p-values, with *P* < 0.05 considered statistically significant. To generate the volcano plot, paired differences in log_10_-transformed metabolite abundances between sleep conditions were calculated for each metabolite within each participant. Statistical comparisons were performed using 2-sided 1-sample tests on these paired differences across participants. Volcano plots were constructed with the x-axis representing the fold-change in metabolite abundance (Normal/Restricted) and the y-axis representing -log_10_(p).

For statistical rhythmicity assessment, raw abundance values for the 90 resulting metabolites were normalized to the mean 24-hr abundance, centering the data on 1. Data were centered based on individual participant means. Each individual metabolite profile was expressed as a percentage of the individual 24-hr mean concentration. These individual normalized profiles were then averaged to illustrate the mean 24-hr profile for each metabolite for the group of participants. Following normalization, eJTK (35) was used to identify rhythmic metabolites, where GammaBH < 0.05 was considered significant. Here, a p-value is calculated from the Gamma fit of an empirical null distribution to generate a GammaP, which is then corrected via application of Benjamini-Hochberg false-discovery rate to yield GammaBH. Unlike JTK_CYCLE, which applies direct multiple testing correction, the GammaBH derived from eJTK enhances flexibility to better control for false-positives in noisy, large-scale ‘omics data. While eJTK analysis does not allow for comparison between study conditions regarding differences in characteristic of the waveform, we are able to report whether profiles are considered rhythmic or not in each sleep condition. Future studies will need to be conducted to ascertain whether rhythmic patterns are similar or different between the two conditions.

### Study approval

The study was approved by the Institutional Review Board of The University of Chicago (IRB#16028A, 1/28/2010), registered on ClinicalTrials.gov (NCT00989976), and all participants were compensated for their participation. All participants provided written informed consent in accordance with the Declaration of Helsinki.

### Data availability

Data are available from the corresponding authors upon request. Values for all data points in graphs are reported in the Supporting Data Values file.

## Author contributions

Conceptualization: ECH, VAL, DPJ, EVC, and EBC. Methodology: ECH, EVC, DPJ, SL and VLT. Validation: AMS. Formal analysis: KF, EAC, AMS, SL, ET, MK, VLT, and VAL. Investigation: ECH, SL, and VLT. Resources: DPJ, EBC, and EVC. Data curation: KF, EAC, AMS, SL, MK, VLT, and ECH. writing—original draft preparation: ECH, VAL, KF, AMS, MK. Writing—review and editing: ECH, VAL, KF, EAC, AMS, ET, MK, DPJ, EBC, and EVC. Visualization: KF, MK, EAC, and AMS. Supervision: ECH and VAL. Funding acquisition: ECH, VAL, EVC, and EBC. All authors have read and agreed to the published version of the manuscript.

## Funding support

This work is the result of NIH funding, in whole or in part, and is subject to the NIH Public Access Policy. Through acceptance of this federal funding, the NIH has been given a right to make the work publicly available in PubMed Central.

- KL2RR025000.
- P30DK020595.
- P30DK042086.
- K01DK111785.
- F31DK122714.
- DOD W81XWH-07-2-0071.
- R56DK102872-01A1.
- The University of Chicago GI Research Foundation.

## Supplementary Material

Supplemental data

ICMJE disclosure forms

Supporting data values

## Figures and Tables

**Table 2:**
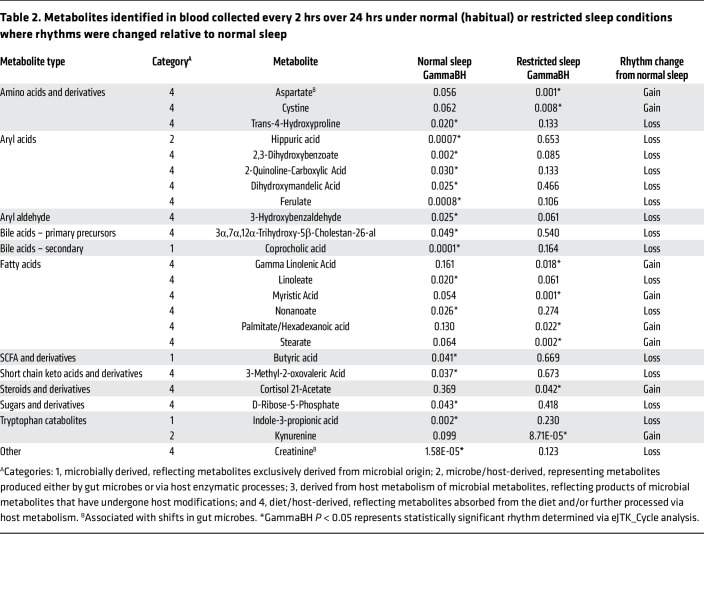
Metabolites identified in blood collected every 2 hrs over 24 hrs under normal (habitual) or restricted sleep conditions where rhythms were changed relative to normal sleep

**Table 1:**
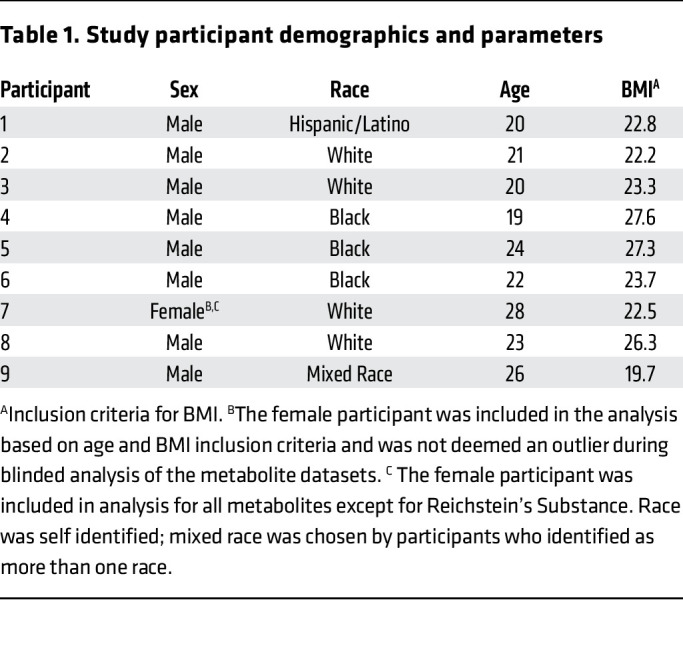
Study participant demographics and parameters

## Version 1 03/16/2026

Electronic publication
