# Supplementary material for: Short-term sleep restriction in humans alters diurnal circulating metabolite profiles, including those of microbial origin
Source: J Clin Invest. 2026 Mar 16;136(6):e189363. doi: 10.1172/JCI189363 (PMC12987657; doi:10.1172/JCI189363)
Supplement: Supplemental data [file jci-136-189363-s301.pdf]

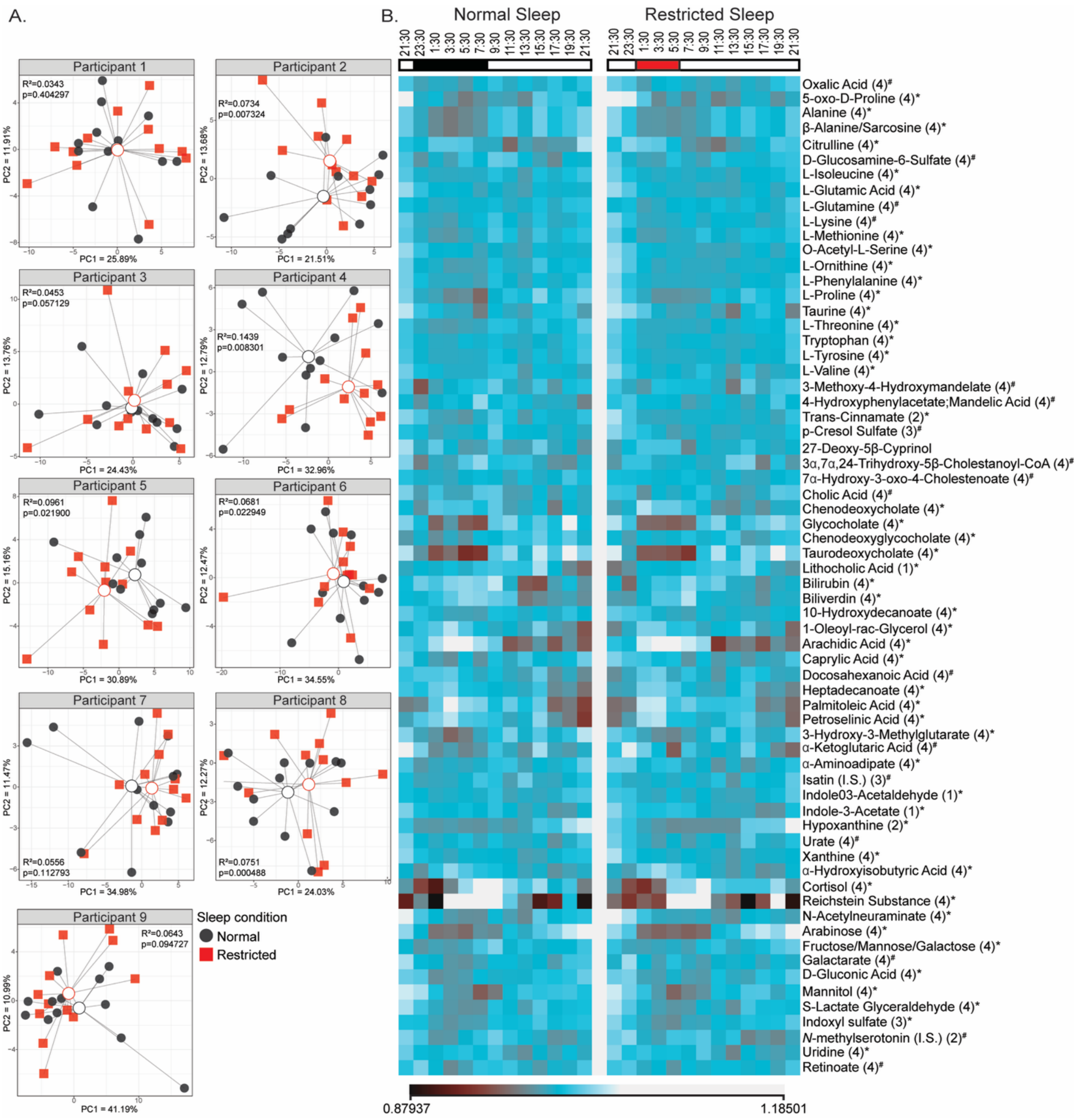

Figure S1. Short-term sleep restriction elicits inter-individual variation in 24-hr metabolite abundances, while rhythmic status remains unchanged in most serum metabolites (**A**) Principal Coordinate Analysis (PCoA) of Euclidian distances calculated from the mean relative abundance over 24-hr of metabolites under normal vs. restricted sleep conditions plotted by individual participant. P-value and pseudo-r^2 determined using PERMANOVA implemented via the adonis2 function (p<0.05 considered statistically significant). (**B**) Heatmap representing mean-

normalized relative abundance of metabolites sampled every 2-hrs over 24-hrs. Black and red bars indicate sleep time in normal and restricted sleep conditions. Metabolite category is listed in parentheses and are as follows: 1 = microbially-derived, reflecting metabolites exclusively derived from microbial origin; 2 = microbe/host-derived, representing metabolites produced either by gut microbes or via host enzymatic processes; 3 = derived from host metabolism of microbial metabolites, reflecting products of microbial metabolites that have undergone host modifications; and 4 = diet/host-derived, reflecting metabolites absorbed from the diet and/or further processed via host metabolism. *Indicates rhythmicity under both normal and sleep restricted conditions and [#] indicates no rhythmicity under both normal and sleep restricted conditions determined via eJTK cycle (GammaBH $p < 0.05$).

Table S1. Metabolites identified in blood collected every 2-hrs over 24-hrs under normal (habitual) or restricted sleep conditions where rhythms were unchanged following the intervention.

Metabolite Type	Category[A]	Metabolite	Normal Sleep GammaBH	Restricted Sleep GammaBH	Rhythm Change from Normal Sleep
Acid	4	Oxalic Acid	0.061	0.068	None
Amino acids and derivatives	4	5-oxo-D-Proline	6.27E-05*	0.0002*	None
	4	Alanine	3.45E-10*	3.35E-08*	None
	4	β-Alanine/Sarcosine	3.45E-10*	3.35E-08*	None
	4	Citrulline	4.52E-05*	3.81E-06*	None
	4	D-Glucosamine-6-Sulfate	0.104	0.147	None
	4	L-Isoleucine	0.0004*	0.006*	None
	4	L-Glutamic Acid	0.008*	7.70E-05*	None
	4	L-Glutamine	0.210	0.136	None
	4	L-Lysine	0.068	0.325	None
	4	L-Methionine	7.99E-05*	0.002*	None
	4	O-Acetyl-L-Serine	0.008*	7.70E-05*	None
	4	L-Ornithine	0.0006*	0.007*	None
	4	L-Phenylalanine	0.0004*	0.0002*	None
	4	L-Proline	3.45E-10*	1.25E-09*	None
	4	Taurine	0.0004*	0.0003*	None
	4	L-Threonine	5.30E-06*	0.0002*	None
	4	Tryptophan	0.031*	0.012*	None
	4	L-Tyrosine	0.005*	0.002*	None
	4	L-Valine	0.351	0.406	None
Aryl acids	4	3-Methoxy-4-Hydroxymandelate	0.163	0.209	None
	4	4-Hydroxyphenylacetate; Mandelic Acid	0.141	0.533	None
	2	Trans-Cinnamate	0.008*	0.011*	None
Aryl Sulfate	3	p-Cresol sulfate	0.248	0.055	None
Bile acids - primary precursors	4	27-Deoxy-5β-Cyprinol	0.248	0.292	None
	4	3α,7α,24-Trihydroxy-5β-Cholestanoyl-CoA	0.383	0.460	None
	4	7α-Hydroxy-3-oxo-4-Cholestenoate	0.097	0.165	None
Bile acids - primary	4	Cholic acid	0.448	0.324	None
	4	Chenodeoxycholate	0.012*	9.50E-08*	None
Bile acids - Glyco-conjugated	4	Glycocholate	3.11E-07*	9.50E-08*	None
	4	Chenodeoxyglycocholate	2.54E-14*	8.95E-12*	None
Bile acids - Tauro-conjugated	4	Taurodeoxycholate	4.52E-14*	7.53E-12*	None
Bile acids - secondary	1	Lithocholic acid	9.16E-07*	4.32E-07*	None
Bile component	4	Bilirubin	8.21E-08*	7.30E-07*	None
	4	Biliverdin	1.01E-08*	7.34E-06*	None
Fatty acids	4	10-Hydroxydecanoate	0.002*	0.004*	None
	4	1-Oleoyl-rac-Glycerol	1.36E-05*	1.28E-05*	None
	4	Arachidic Acid	1.72E-16*	6.00E-21*	None
	4	Caprylic Acid	4.78E-05*	0.0002*	None
	4	Docosahexanoic Acid	0.063	0.141	None
	4	Heptadecanoate	3.20E-05*	1.76E-06*	None
	4	Palmitoleic Acid	1.42E-07*	8.28E-10*	None

	4	Petroselinic Acid	2.11E-05*	1.04E-06*	None
Glutaric acid derivatives	4	3-Hydroxy-3-Methylglutarate	1.82E-08*	0.0003*	None
	4	α-Ketoglutaric Acid	0.064	0.098	None
Homoisocitrate derivative	4	α-Aminoadipate	0.010*	0.002*	None
Indoles and derivatives	3	Isatin (I.S.)	0.532	0.771	None
	1	Indole-3-Acetaldehyde	0.018*	0.018*	None
	1	Indole-3-Acetate	0.001*	1.53E-07*	None
Purines and derivatives	2	Hypoxanthine	2.29E-08*	5.29E-09*	None
	4	Urate	0.071	0.085	None
	4	Xanthine	0.006*	0.0003*	None
SCFA and derivatives	4	α-Hydroxyisobutyric Acid	1.72E-06*	1.22E-10*	None
Steroids and derivatives	4	Cortisol	7.09E-20*	8.06E-14*	None
	4	Reichstein Substance	1.59E-07*	0.0007*	None
Sugars and derivatives	4	N-Acetylneuraminate	2.02E-05*	1.29E-05*	None
	4	Arabinose	1.32E-14*	1.24E-14*	None
	4	Fructose/Mannose/Galactose	0.010*	0.0002*	None
	4	Galactarate	0.064	0.393	None
	4	D-Gluconic Acid	0.001*	0.009*	None
	4	Mannitol	0.001*	0.002*	None
	4	S-Lactate-Glyceraldehyde	3.01E-07*	3.35E-08*	None
Tryptophan catabolites	3	Indoxyl sulfate	6.56E-06*	4.92E-12*	None
	2	*N*-methylserotonin (I.S.)	0.532	0.080	None
Uracil containing precursor and derivatives	4	Uridine	8.90E-05*	0.0002*	None
Vitamin	4	Retinoate	0.161	0.596	None

[A]Categories: 1 = microbially-derived, reflecting metabolites exclusively derived from microbial origin; 2 = microbe/host-derived, representing metabolites produced either by gut microbes or via host enzymatic processes; 3 = derived from host metabolism of microbial metabolites, reflecting products of microbial metabolites that have undergone host modifications; and 4 = diet/host-derived, reflecting metabolites absorbed from the diet and/or further processed via host metabolism. *GammaBH $p < 0.05$ represents statistically significant rhythm determined via eJTK_Cycle analysis.

Table S2. Detected metabolites mapped to Homo sapien (hsa) KEGG pathways*. Of the 90 compounds searched, 71 compounds** mapped to at least 1 hsa pathway***. Yellow, always rhythmic; Green, gained rhythms from normal sleep; Red, lost rhythms from normal sleep; Grey, never rhythmic. Bolded metabolites are microbially-associated.

KEGG Pathway (122)

RHYTHMIC STATUS	Common Metabolite Name	KEGG ID	2-Oxocarboxylic acid metabolism - hsa01210	ABC transporters - hsa02010	African trypanosomiasis - hsa5143	Alanine, aspartate and glutamate metabolism - hsa00250	Alcoholism - hsa05034	Aldosterone-regulated sodium reabsorption - hsa04960	Aldosterone synthesis and secretion - hsa04925	Amino sugar and nucleotide sugar metabolism - hsa00520	Aminoacyl-tRNA biosynthesis - hsa00970	Amphetamine addiction - hsa05031	Amyotrophic lateral sclerosis - hsa05014	Arginine and proline metabolism - hsa00330	Antifolate resistance - hsa01523	Arginine biosynthesis - hsa00220	Ascorbate and aldarate metabolism - hsa00053	betaAlanine metabolism - hsa00410	Bile secretion - has04976	Biosynthesis of amino acids - hsa01230	Biosynthesis of cofactors - hsa01240	Biosynthesis of nucleotide sugars - hsa01250	Biosynthesis of unsaturated fatty acids - hsa01040	Butanoate metabolism - hsa00650	Biosynthesis of nucleotide sugars - hsa01250	Biotin metabolism - hsa00780	C-type lectin receptor signaling pathwa -	Caffeine metabolism - hsa00232	Carbohydrate digestion and absorption -	Carbon metabolism - hsa01200	Central carbon metabolism in cancer - hsa05230	Cholesterol metabolism - hsa04979
	NUMBER OF METABOLITES WITHIN EACH PATHWAY		13	17	2	5	2	1	1	3	13	2	1	5	1	5	3	2	9	19	12	1	6	3	2	1	1	1	2	7	12	2
ALWAYS	5-OXO-D-PROLINE; 5-OXO-L-PROLINE	C01879																														
	ALPHA-AMINOADIPATE; N-METHYLLGLUTAMATE	C00956	X																X													
	ARABINOSE	C00259		X						X						X								X							
	ARACHIDIC ACID	C06425																						X									
	BETA-ALANINE; SARCOSINE	C00099																		X			X												
	BILIRUBIN	C00486																			X														
	BILIVERDIN	C00500																														
	CAPRYLIC ACID	C06423																					X												
	CHENODEOXYCHOLATE	C02528																			X														
	CHENODEOXYGLYCOCHOLATE	C05466																			X														X
	CITRULLINE	C00327																X				X												
	CORTISOL	C00735						X													X														
	FRUCTOSE/MANNOSE/GALACTOSE****	C00095/ C00159/ C00124		X						X												X					X		X			
	GLYCOCHOLATE	C01921																			X														X
	**HYPOXANTHINE**	**C00262**																														
	**INDOLE-3-ACETALDEHYDE**	**C00637**																														
	**INDOLE-3-ACETATE; INDOLE-3-ACETIC ACID**	**C00954**																														
	L-ALANINE	C00041	X	X		X					X											X										X	X	
	L-GLUTAMATE/L-GLUTAMIC ACID	C00025	X	X		X	X				X	X	X	X		X				X	X			X						X	X	
	L-ISOLEUCINE	C00407	X	X							X											X											X	
	L-METHIONINE	C00073	X								X				X					X	X													X	
	L-ORNITHINE	C00515																														
	L-PHENYLALANINE	C00079	X	X							X											X											X	
	L-PROLINE	C00148		X							X			X						X											X	
	L-THREONINE	C00188		X							X										X												
	L-TYROSINE	C00082	X				X				X	X								X	X											X	
	**LITHOCHOLIC ACID**	**C03990**																			X														
	MANNITOL	C00392		X																												
	N-ACETYLNEURAMINATE	C00270								X																	X							
	O-ACETYL-L-SERINE	C00979																		X										X		
	PALMITOLEIC ACID	C08362																														
	REICHSTEIN SUBSTANCE	C02140							X																								
	TAURINE	C00245		X																												
	TRYPTOPHAN	C00078	X		X						X			X						X	X											X	
	URIDINE	C00299		X																												
	XANTHINE	C00385																											X				
NEVER	3-METHOXY-4-HYDROXY-MANDELATE	C05584																														
	3α,7α,24-TRIHYDROXY-5β-CHOLESTANOYL-COA	C05448																														
	27-DEOXY-5β-CYPRINOL	C05446																														
	7α-HYDROXY-3-OXO-4-CHOLESTENOATE	C17337																														
	4-HYDROXY-PHENYLACETATE/MANDELIC ACID	C00642																														
	α-KETOGLUTARIC ACID	C00026	X			X											X	X		X	X	X				X						X	X	
	CHOLIC ACID	C00695																			X														
	DOCOSAHEXAENOIC ACID	C06429																						X									
	GALACTARATE	C00879																	X																
	L-GLUTAMINE	C00064		X		X					X					X				X	X										X	
	L-LYSINE	C00047	X	X							X										X						X							
	L-VALINE	C00183	X	X							X										X	X											X	
	N-METHYLSEROTONIN	C06212																														
	OXALIC ACID	C00209																														
	RETINOATE	C00777																														
	**TRANS-CINNAMATE**	**C00423**																														
	URATE	C00366																			X														
GAINED	GAMMA LINOLENIC ACID	C06426																						X									
	HEXADECANOIC ACID/PALMITATE	C00249																						X									
	**KYNURENINE**	**C00328**			X																	X												
	L-ASPARTATE	C00049	X	X		X					X					X		X		X	X											X	X	
	L-CYSTINE	C00491		X																												
	MYRISTIC ACID	C06424																														
	STEARATE	C01530																						X									
LOST	(S)3-METHYL-2-OXOVALERIC ACID	C00671	X																		X												
	3α,7α,12α-TRIHYDROXY-5β-CHOLESTAN-26-AL	C01301																														
	**BUTYRIC ACID**	**C00246**																							X					X			
	COPROCHOLIC ACID	C04722																														
	**CREATININE**	**C00791**												X																			
	D-GLUCONIC ACID	C00257																												X		
	DIHYDROXYMANDELIC ACID	C05580																														
	D-RIBOSE-5-PHOSPHATE	C00117																		X	X										X		
	FERULATE	C01494																														
	LINOLEATE	C01595																						X									
	TRANS-4-HYDROXYPROLINE; 4-HYDROXY-L-PROLINE; N-ACETYL-L-ALANINE; CIS-4-HYDROXY-D-PROLINE	C01157		X										X																			

RHYTHMIC STATUS	Common Metabolite Name	KEGG ID	Circadian entrainment - hsa04713	Citrate cycle (TCA cycle) - hsa00020	Cobalamin transport and metabolism - hsa04980	Cocaine addiction - hsa05030	Cortisol synthesis and secretion - hsa04927	Cushing syndrome - hsa04934	Cysteine and methionine metabolism - hsa00270	Diabetic cardiomyopathy - hsa05415	D+H:DK-amino acid metabolism - hs00470	Dopaminergic synapse - hsa04728	Efferocytosis - hsa04148	Fatty acid biosynthesis - hsa00061	Fatty acid degradation - hsa00071	Fatty acid elongation - has00062	Fatty acid metabolism - hsa01212	Ferroptosis -hsa04216	Fox0 signalling pathway - hsa04068	Fructose and mannose metabolism - hsa00051	GABAergic synapse - hsa04727	Galactose metabolism - hsa00052	Gap junction - hsa04540	Gastric cancer - hsa05226	Glucagon signaling pathway - hsa04922	Glutamatergic synapse - hsa04724	Glutathione metabolism - hsa00480	Glycine, serine and threonine metabolism - hsa00260	Glyoxylate and dicarboxylate metabolism - hsa00630	HIF1 signaling pathway - hsa04066	Histidine metabolism - hsa00340	Huntington disease -hsa05016
	NUMBER OF METABOLITES WITHIN EACH PATHWAY		1	1	1	2	1	1	5	1	12	1	1	5	1	1	1	2	1	2	3	1	1	1	1	2	2	3	4	1	3	1
ALWAYS	5-OXO-D-PROLINE; 5-OXO-L-PROLINE	C01879																								X					
	ALPHA-AMINOADIPATE; N-METHYLLGLUTAMATE	C00956																														
	ARABINOSE	C00259																														
	ARACHIDIC ACID	C06425																														
	BETA-ALANINE; SARCOSINE	C00099																														
	BILIRUBIN	C00486																														
	BILIVERDIN	C00500																														
	CAPRYLIC ACID	C06423												X																	
	CHENODEOXYCHOLATE	C02528																														
	CHENODEOXYGLYCOCHOLATE	C05466																														
	CITRULLINE	C00327																														
	CORTISOL	C00735					X	X																								
	FRUCTOSE/MANNOSE/GALACTOSE****	C00095/ C00159/ C00124								X									X		X										
	GLYCOCHOLATE	C01921																														
	**HYPOXANTHINE**	**C00262**																														
	**INDOLE-3-ACETALDEHYDE**	**C00637**																														
	**INDOLE-3-ACETATE; INDOLE-3-ACETIC ACID**	**C00954**																														
	L-ALANINE	C00041							X		X																						
	L-GLUTAMATE/L-GLUTAMIC ACID	C00025	X			X					X						X	X		X		X			X	X		X			X
	L-ISOLEUCINE	C00407																														
	L-METHIONINE	C00073			X				X		X		X																			
	L-ORNITHINE	C00515									X																				
	L-PHENYLALANINE	C00079									X																				
	L-PROLINE	C00148									X																				
	L-THREONINE	C00188									X																X				
	L-TYROSINE	C00082				X						X																			
	**LITHOCHOLIC ACID**	**C03990**																														
	MANNITOL	C00392																		X											
	N-ACETYLNEURAMINATE	C00270																														
	O-ACETYL-L-SERINE	C00979							X																							
	PALMITOLEIC ACID	C08362												X																	
	REICHSTEIN SUBSTANCE	C02140																														
	TAURINE	C00245																														
	TRYPTOPHAN	C00078																										X				
	URIDINE	C00299																														
	XANTHINE	C00385																														
NEVER	3-METHOXY-4-HYDROXY-MANDELATE	C05584																														
	3α,7α,24-TRIHYDROXY-5β-CHOLESTANOYL-COA	C05448																														
	27-DEOXY-5β-CYPRINOL	C05446																														
	7α-HYDROXY-3-OXO-4-CHOLESTENOATE	C17337																														
	4-HYDROXY-PHENYLACETATE/MANDELIC ACID	C00642																														
	α-KETOGLUTARIC ACID	C00026		X							X									X			X				X	X	X	
	CHOLIC ACID	C00695																														
	DOCOSAHEXAENOIC ACID	C06429																														
	GALACTARATE	C00879																														
	L-GLUTAMINE	C00064									X										X					X			X		X	
	L-LYSINE	C00047									X																				
	L-VALINE	C00183																														
	N-METHYLSEROTONIN	C06212																														
	OXALIC ACID	C00209																										X			
	RETINOATE	C00777																						X								
	**TRANS-CINNAMATE**	**C00423**																														
	URATE	C00366																														
GAINED	GAMMA LINOLENIC ACID	C06426																														
	HEXADECANOIC ACID/PALMITATE	C00249												X	X	X	X															
	**KYNURENINE**	**C00328**																														
	L-ASPARTATE	C00049							X		X																X			X	
	L-CYSTINE	C00491							X									X													
	MYRISTIC ACID	C06424												X																	
	STEARATE	C01530												X																	
LOST	(S)3-METHYL-2-OXOVALERIC ACID	C00671																														
	3α,7α,12α-TRIHYDROXY-5β-CHOLESTAN-26-AL	C01301																														
	**BUTYRIC ACID**	**C00246**																														
	COPROCHOLIC ACID	C04722																														
	**CREATININE**	**C00791**																														
	D-GLUCONIC ACID	C00257																														
	DIHYDROXYMANDELIC ACID	C05580																														
	D-RIBOSE-5-PHOSPHATE	C00117																														
	FERULATE	C01494																														
	LINOLEATE	C01595																														
	TRANS-4-HYDROXYPROLINE; 4-HYDROXY-L-PROLINE; N-ACETYL-L-ALANINE; CIS-4-HYDROXY-D-PROLINE	C01157									X																				

RHYTHMIC STATUS	Common Metabolite Name	KEGG ID	Insulin resistance - hsa04931	Intestinal immune network for IgA production - hsa04672	Linoleic acid metabolism - hsa00591	Lipoic acid metabolism - hsa00785	Long-term depression - hsa04730	Long-term potentiation - hsa04720	Lysine degradation - hsa00310	Lysosome - hsa04142	Melanogenesis - hsa04916	Metabolic pathway - hsa01100	Mineral absorption - hsa04978	Neomycin, kanamycin, and gentamicin biosynthesis - hsa00524	Neuroactive ligand receptor interaction -	Nicotinate and nicotinamide metabolism -	Nicotine addiction - hsa05033	Nitrogen Metabolism - 00910	Non-small cell lung cancer - hsa05223	Nucleotide metabolism - hsa01232	Pantothenate and CoA biosynthesis - hsa00770	Parkinson disease - hsa05012	Pathways in cancer - hsa05200	Pathways of neurodegeneration - hsa05022	Pentose and glucuronate interconversions - hsa00040	Pentose phosphate pathway - hsa00030	Phenylalanine metabolism - hsa00360	Phenylalanine, tyrosine and tryptophan biosynthesis - hsa00400	Phospholipase D signaling pathway - hsa04072	Phosphonate and phosphinate metabolism - hsa00440	Porphyrin and chlorophyll metabolism -	Primary bile acid biosynthesis - hsa00120
	NUMBER OF METABOLITES WITHIN EACH PATHWAY		1	1	2	3	1	1	3	1	1	55	10	1	5	1	1	2	1	4	3	1	2	1	2	2	4	3	1	1	4	10
ALWAYS	5-OXO-D-PROLINE; 5-OXO-L-PROLINE	C01879										X																				
	ALPHA-AMINOADIPATE; N-METHYLLGLUTAMATE	C00956							X			X																				
	ARABINOSE	C00259										X													X							
	ARACHIDIC ACID	C06425																														
	BETA-ALANINE; SARCOSINE	C00099										X			X						X											
	BILIRUBIN	C00486										X																			X	
	BILIVERDIN	C00500										X																			X	
	CAPRYLIC ACID	C06423				X						X																				
	CHENODEOXYCHOLATE	C02528										X																				X
	CHENODEOXYGLYCOCHOLATE	C05466																														X
	CITRULLINE	C00327										X																				
	CORTISOL	C00735										X			X								X										
	FRUCTOSE/MANNOSE/GALACTOSE****	C00095/ C00159/ C00124	X								X		X	X																				
	GLYCOCHOLATE	C01921										X																				X
	**HYPOXANTHINE**	**C00262**										X								X													
	**INDOLE-3-ACETALDEHYDE**	**C00637**										X																				
	**INDOLE-3-ACETATE; INDOLE-3-ACETIC ACID**	**C00954**										X																				
	L-ALANINE	C00041										X	X																			
	L-GLUTAMATE/L-GLUTAMIC ACID	C00025					X	X				X		X	X		X	X							X					X		X	
	L-ISOLEUCINE	C00407										X	X																			
	L-METHIONINE	C00073										X	X																			
	L-ORNITHINE	C00515										X																				
	L-PHENYLALANINE	C00079										X	X													X	X				
	L-PROLINE	C00148										X	X																			
	L-THREONINE	C00188										X	X																	X	
	L-TYROSINE	C00082									X	X										X					X	X				
	**LITHOCHOLIC ACID**	**C03990**																														
	MANNITOL	C00392										X																				
	N-ACETYLNEURAMINATE	C00270										X																				
	O-ACETYL-L-SERINE	C00979										X																				
	PALMITOLEIC ACID	C08362										X																				
	REICHSTEIN SUBSTANCE	C02140										X																				
	TAURINE	C00245										X			X																		X
	TRYPTOPHAN	C00078										X	X																X				
	URIDINE	C00299										X								X													
	XANTHINE	C00385										X								X													
NEVER	3-METHOXY-4-HYDROXY-MANDELATE	C05584										X																				
	3α,7α,24-TRIHYDROXY-5β-CHOLESTANOYL-COA	C05448										X																				X
	27-DEOXY-5β-CYPRINOL	C05446										X																				X
	7α-HYDROXY-3-OXO-4-CHOLESTENOATE	C17337																														X
	4-HYDROXY-PHENYLACETATE/MANDELIC ACID	C00642										X														X					
	α-KETOGLUTARIC ACID	C00026				X			X			X													X							
	CHOLIC ACID	C00695										X																				X
	DOCOSAHEXAENOIC ACID	C06429										X																				
	GALACTARATE	C00879										X																				
	L-GLUTAMINE	C00064										X	X					X		X													
	L-LYSINE	C00047							X			X																				
	L-VALINE	C00183										X	X								X											
	N-METHYLSEROTONIN	C06212																														
	OXALIC ACID	C00209										X																				
	RETINOATE	C00777		X								X							X				X									
	**TRANS-CINNAMATE**	**C00423**										X														X					
	URATE	C00366										X																				
GAINED	GAMMA LINOLENIC ACID	C06426			X							X																				
	HEXADECANOIC ACID/PALMITATE	C00249										X																				
	**KYNURENINE**	**C00328**										X																				
	L-ASPARTATE	C00049										X			X	X					X											
	L-CYSTINE	C00491										X																				
	MYRISTIC ACID	C06424										X																				
	STEARATE	C01530										X																				
LOST	(S)3-METHYL-2-OXOVALERIC ACID	C00671				X						X																				
	3α,7α,12α-TRIHYDROXY-5β-CHOLESTAN-26-AL	C01301										X																				X
	**BUTYRIC ACID**	**C00246**										X																				
	COPROCHOLIC ACID	C04722										X																				X
	**CREATININE**	**C00791**										X																				
	D-GLUCONIC ACID	C00257										X													X						
	DIHYDROXYMANDELIC ACID	C05580										X																				
	D-RIBOSE-5-PHOSPHATE	C00117										X													X				X		
	FERULATE	C01494										X																				
	LINOLEATE	C01595			X							X																				
	TRANS-4-HYDROXYPROLINE; 4-HYDROXY-L-PROLINE; N-ACETYL-L-ALANINE; CIS-4-HYDROXY-D-PROLINE	C01157										X																				

RHYTHMIC STATUS	Common Metabolite Name	KEGG ID	Prion disease - hsa05020	Propanoate metabolism - hsa00640	Prolactin signaling pathway - hsa04917	Prostate cancer - hsa05215	Protein digestion and absorption - hsa04974	Proximal tubule bicarbonate reclamation - hsa04964	Purine metabolism - hsa00230	Pyrimidine metabolism - hsa00240	Regulation of lipolysis in adipocytes - hsa04923	Retinol metabolism - hsa00830	Retrograde endocannabinoid signaling -	Selenocompound metabolism - hsa00450	Serotonergic synapse - hsa04726	Shigellosis - hsa05131	Small cell lung cancer - hsa05222	Spinocerebellar ataxia - hsa05017	Starch and sucrose metabolism - hsa00500	Steroid hormone biosynthesis - hsa00140	Sulfur metabolism - hsa00920	Sulfur relay system - hsa04122	Synaptic vesicle cycle - hsa04721	Taste transduction - hsa04742	Taurine and hypotaurine metabolism - hsa00430	Th17 cell differentiation - hsa04659	Thiamine metabolism - hsa00730	Thyroid hormone synthesis - hsa04918	Tryptophan metabolism - hsa00380	Tyrosine metabolism - hsa00350	Ubiquinone and other terpenoid-quinone biosynthesis - hsa00130	Valine, leucine and isoleucine biosynthesis - hsa00280	Valine, leucine, and isoleucine degradation - hsa00290	Vitamin B6 metabolism - hsa00750
	NUMBER OF METABOLITES WITHIN EACH PATHWAY		1	1	1	1	16	3	6	3	1	1	1	1	1	1	1	1	1	2	2	2	1	2	4	1	1	1	5	4	2	4	3	2
ALWAYS	5-OXO-D-PROLINE; 5-OXO-L-PROLINE	C01879																																
	ALPHA-AMINOADIPATE; N-METHYLLGLUTAMATE	C00956																																
	ARABINOSE	C00259																																
	ARACHIDIC ACID	C06425																																
	BETA-ALANINE; SARCOSINE	C00099		X			X			X																									
	BILIRUBIN	C00486																																
	BILIVERDIN	C00500																																
	CAPRYLIC ACID	C06423																																
	CHENODEOXYCHOLATE	C02528																																
	CHENODEOXYGLYCOCHOLATE	C05466																																
	CITRULLINE	C00327																																
	CORTISOL	C00735				X													X														
	FRUCTOSE/MANNOSE/GALACTOSE****	C00095/ C00159/ C00124																	X					X										
	GLYCOCHOLATE	C01921																																
	**HYPOXANTHINE**	**C00262**							X																										
	**INDOLE-3-ACETALDEHYDE**	**C00637**																											X						
	**INDOLE-3-ACETATE; INDOLE-3-ACETIC ACID**	**C00954**																											X						
	L-ALANINE	C00041					X						X								X			X											
	L-GLUTAMATE/L-GLUTAMIC ACID	C00025					X	X					X					X					X	X	X											
	L-ISOLEUCINE	C00407					X								X															X	X		
	L-METHIONINE	C00073					X																												
	L-ORNITHINE	C00515																																
	L-PHENYLALANINE	C00079					X																												
	L-PROLINE	C00148					X																												
	L-THREONINE	C00188					X																								X				
	L-TYROSINE	C00082			X		X																		X			X	X				
	**LITHOCHOLIC ACID**	**C03990**																																
	MANNITOL	C00392																																
	N-ACETYLNEURAMINATE	C00270																																
	O-ACETYL-L-SERINE	C00979																				X	X													
	PALMITOLEIC ACID	C08362																																
	REICHSTEIN SUBSTANCE	C02140	X								X								X														
	TAURINE	C00245																				X			X											
	TRYPTOPHAN	C00078					X								X														X						
	URIDINE	C00299								X																								
	XANTHINE	C00385							X																										
NEVER	3-METHOXY-4-HYDROXY-MANDELATE	C05584																												X					
	3α,7α,24-TRIHYDROXY-5β-CHOLESTANOYL-COA	C05448																																
	27-DEOXY-5β-CYPRINOL	C05446																																
	7α-HYDROXY-3-OXO-4-CHOLESTENOATE	C17337																																
	4-HYDROXY-PHENYLACETATE/MANDELIC ACID	C00642																												X					
	α-KETOGLUTARIC ACID	C00026						X																X											
	CHOLIC ACID	C00695																																
	DOCOSAHEXAENOIC ACID	C06429																																
	GALACTARATE	C00879																																
	L-GLUTAMINE	C00064					X	X	X	X																										X
	L-LYSINE	C00047					X																												
	L-VALINE	C00183					X																										X	X		
	N-METHYLSEROTONIN	C06212																											X						
	OXALIC ACID	C00209							X																										
	RETINOATE	C00777										X				X								X										
	**TRANS-CINNAMATE**	**C00423**																													X				
	URATE	C00366							X																										
GAINED	GAMMA LINOLENIC ACID	C06426																																
	HEXADECANOIC ACID/PALMITATE	C00249																																
	**KYNURENINE**	**C00328**																											X						
	L-ASPARTATE	C00049					X																												
	L-CYSTINE	C00491					X																												
	MYRISTIC ACID	C06424																																
	STEARATE	C01530																																
LOST	(S)3-METHYL-2-OXOVALERIC ACID	C00671																														X	X		
	3α,7α,12α-TRIHYDROXY-5β-CHOLESTAN-26-AL	C01301																																
	**BUTYRIC ACID**	**C00246**					X																												
	COPROCHOLIC ACID	C04722																																
	**CREATININE**	**C00791**																																
	D-GLUCONIC ACID	C00257																																
	DIHYDROXYMANDELIC ACID	C05580																												X					
	D-RIBOSE-5-PHOSPHATE	C00117							X																			X							X
	FERULATE	C01494																																
	LINOLEATE	C01595																																
	TRANS-4-HYDROXYPROLINE; 4-HYDROXY-L-PROLINE; N-ACETYL-L-ALANINE; CIS-4-HYDROXY-D-PROLINE	C01157																																

*p-cresol sulfate, 1-oleoyl-rac-glycerol, heptadecanoate, alpha-hydroxyisobutyric acid, and indoxyl sulfate do not have KEGG IDs and were excluded from the table.

**2,3-Dihydroxybenzoate (C00196) and 3-Hydroxybenzaldehyde (C03067) do not map to any hsa pathways.

***The following compounds have KEGG IDs but do not map to hsa KEGG pathways: D-glucosamine-6 sulfate (C04132), 2-quinoline-carboxylic acid (C06325), Taurodeoxycholate (C05463) 10-Hydroxydecanoate (C02774), Noanoate (C01601); Petroselinic acid (C08363), 3-Hydroxy-3-methylglutarate (C03761), Indole-3-propionic acid (C22236), Isatin (C11129), Cortisol-21-Acetate (C02821)

****Fructose, Mannose, and Galactose exhibit identical MS fragments that could not be resolved at the individual compound level

Table S3. Detected metabolites mapped to KEGG pathways other than those designated to Homo sapien (hsa). Of the 90 compounds searched, 46 compounds mapped to at least one non-hsa pathway. Yellow, always rhythmic; Green, gained rhythms from normal sleep; Red, lost rhythms from normal sleep; Grey, never rhythmic. Bolded metabolites are microbially-associated.

			KEGG Pathway (54)																																																					
	**Common Metabolite Name**	**KEGG ID**	**Aminobenzoate degradation - map01053**	**Arginine biosynthesis - map00220**	**Axon regeneration - map04361**	**Bacterial chemotaxis - map02030**	**Benzoate degradation - map00362**	**Betalain biosynthesis - map00965**	**Biosynthesis of alkaloids derived from histidine and purine - map01065**	**Biosynthesis of alkaloids derived from ornithine, lysine and nicotinic acid - map01064**	**Biosynthesis of alkaloids derived from shikimate pathway - map01063**	**Biosynthesis of alkaloids derived from terpenoid and polyketide - map01066**	**Biosynthesis of enediyne antibiotics - map01059**	**Biosynthesis of phenylpropanoids - map01061**	**Biosynthesis of plant hormones - map01070**	**Biosynthesis of plant secondary metabolites - map01060**	**Biosynthesis of secondary metabolites -**	**Biosynthesis of siderophore group nonribosomal peptides - map01053**	**Biosynthesis of terpenoids and steroids -**	**Biosynthesis of vancomycin group antibiotics - map01055**	**Biosynthesis of various antibiotics - map00998**	**Biosynthesis of various alkaloids - map00996**	**Biosynthesis of various other secondary metabolites - map00997**	**Biosynthesis of various plant secondary metabolites - map00999**	**C5-Branched dibasic acid metabolism -**	**Carbapenem biosynthesis - map00332**	**Carbon fixation by Calvin Cycle - map00710**	**Chloroalkane and chloroalkene degradation - map00625**	**Cutin, suberine and wax biosynthesis - map00073**	**Cyanoamino acid metabolism - map00460**	**Degradation of aromatic compounds - map01220**	**Degradation of flavenoids - map00946**	**Furfural degradation - map00365**	**Glucocorticoid and mineralocorticoid receptor agonsits/antagonsits - map07225**	**Glucosinolate biosynthesis - map00966**	**Indole alkaloid biosynthesis - map00901**	**Isoquinoline alkaloid biosynthesis - map00950**	**Lysine biosynthesis - map00300**	**Methane metabolism - map00680**	**Microbial metabolism in diverse environments - map01120**	**Monobactam biosynthesis - map00261**	**Novobiocin biosynthesis - map00401**	**O-Antigen nucleotide sugar biosynthesis -**	**Other carbon fixation pathways - map00720**	**Phenylpropanoid biosynthesis - map00940**	**Phosphotransferase system (PTS) - map02060**	**Phototransduction - fly - map04745**	**Penicillin and cephalosporin biosynthesis - map00311**	**Plant hormone signal transduction - map04075**	**Prodigiosin biosynthesis - map00333**	**Secondary bile acid biosynthesis - map00121**	**Staurosporine biosynthesis - map00404**	**Tropane, piperidine and pyridine alkaloid biosynthesis - map00960**	**Toluene degradation - map00623**	**Two-component system -map02020**	**Zeatin biosynthesis - map00908**
**RHYTHMIC STATUS**	NUMBER OF METABOLITES WITHIN EACH PATHWAY		2	1	1	2	1	1	2	5	5	3	1	6	7	18	#	1	1	1	5	3	9	6	2	2	2	1	1	5	3	1	1	1	7	1	1	3	1	19	3	2	1	1	4	2	1	2	1	1	5	2	3	2	2	1
**ALWAYS**	ALPHA-AMINOADIPATE; N-METHYLLGLUTAMATE	C00956	X														X																							X								X								
	BETA-ALANINE; SARCOSINE	C00099															X																																							
	BILIRUBIN	C00486															X																																							
	BILIVERDIN	C00500															X																																							
	CAPRYLIC ACID	C06423										X		X																																										
	CHENODEOXYCHOLATE	C02528																																																	X					
	CHENODEOXYGLYCOCHOLATE	C05466																																																	X					
	CITRULLINE	C00327															X																																							
	CORTISOL	C00735																																X																						
	FRUCTOSE/MANNOSE/GALACTOSE*	C00095/ C00159/ C00124				X											X																							X						X								X		
	GLYCOCHOLATE	C01921																																																	X					
	**INDOLE-3-ACETATE; INDOLE-3-ACETIC ACID**	C00954													X																																		X							
	L-ALANINE	C00041														X	X						X																	X																
	L-GLUTAMATE/L-GLUTAMIC ACID	C00025								X						X	X				X		X		X	X														X															X	
	L-ISOLEUCINE	C00407										X				X	X													X					X																		X			
	L-METHIONINE	C00073													X	X	X							X											X																					
	L-ORNITHINE	C00515																																																						
	L-PHENYLALANINE	C00079								X	X			X	X	X	X					X	X	X						X					X										X								X			
	L-PROLINE	C00148															X									X																X								X		X				
	L-THREONINE	C00188														X	X																							X	X															
	L-TYROSINE	C00082						X			X		X			X	X			X	X		X	X						X					X		X		X		X	X			X											
	**LITHOCHOLIC ACID**	C03990																																																	X					
	MANNITOL	C00392																																												X										
	N-ACETYLNEURAMINATE	C00270																																									X													
	O-ACETYL-L-SERINE	C00979															X				X																			X																X
	TRYPTOPHAN	C00078			X						X			X	X	X	X					X	X	X											X	X																X				
	XANTHINE	C00385							X							X	X																							X																
**NEVER**	4-HYDROXY-PHENYLACETATE/MANDELIC ACID	C00642																													X	X								X																
	α-KETOGLUTARIC ACID	C00026							X	X	X	X		X	X	X	X		X		X		X		X								X					X		X				X												
	CHOLIC ACID	C00695																																																	X					
	L-GLUTAMINE	C00064														X	X																							X																
	L-LYSINE	C00047								X						X	X						X															X		X													X			
	L-VALINE	C00183									X					X	X													X					X													X								
	OXALIC ACID	C00209																										X												X																
	**TRANS-CINNAMATE**	C00423													X	X	X					X		X							X									X					X											
	URATE	C00366																																						X																
**GAINED**	HEXADECANOIC ACID/PALMITATE	C00249														X													X																											
	L-ASPARTATE	C00049		X		X				X					X	X	X				X		X	X			X			X								X		X	X														X	
	STEARATE	C01530														X																															X									
**LOST**	2,3-DIHYDROXYBENZOATE	C00196	X				X							X			X	X																						X																
	(S)3-METHYL-2-OXOVALERIC ACID	C00671																																	X																					
	3-HYDROXYBENZALDEHYDE	C03067																													X									X														X		
	D-GLUCONIC ACID	C00257															X																							X																
	D-RIBOSE-5-PHOSPHATE	C00117															X										X													X																
	FERULATE	C01494												X			X						X																						X											
	LINOLEATE	C01595														X																																								

*Fructose, Mannose, Galactose exhibit identical MS fragments that were not able to be resolved at the individual compound level

Table S4. Metabolites identified in blood collected every 2 hrs over a 24 hr period under normal (habitual) or restricted sleep conditions and corresponding eJTK_cycle rhythms analyses, including time of max and min values, max and min values, and max amplitude.

Metabolite	Time of Max		Time of Min		Max Value		Min Value		Max Amplitude	
	Normal	*Restricted*	*Normal*	*Restricted*	*Normal*	*Restricted*	*Normal*	*Restricted*	*Normal*	*Restricted*
Oxalic Acid	9:30	21:30	15:30	9:30	1.045 ± 0.008	1.048 ± 0.01	0.950 ± 0.006	0.953 ± 0.008	0.094 ± 0.014	0.095 ± 0.012
5-oxo-D-Proline	21:30	21:30	23:30	7:30	1.10 ± 0.014	1.109 ± 0.014	0.947 ± 0.007	0.942 ± 0.006	0.15 ± 0.02	0.168 ± 0.017
Alanine	21:30	21:30	7:30	5:30	1.045 ± 0.004	1.045 ± 0.008	0.957 ± 0.003	0.970 ± 0.003	0.088 ± 0.006	0.076 ± 0.009
Aspartate	9:30	21:30	3:30	5:30	1.04 ± 0.004	1.039 ± 0.009	0.975 ± 0.002	0.973 ± 0.002	0.065 ± 0.006	0.066 ± 0.01
β-Alanine/Sarcosine	21:30	21:30	7:30	5:30	1.045 ± 0.004	1.045 ± 0.008	0.957 ± 0.003	0.969 ± 0.003	0.088 ± 0.006	0.076 ± 0.009
Citrulline	3:30	23:30	1:30	21:30	1.062 ± 0.011	1.057 ± 0.006	0.939 ± 0.008	0.943 ± 0.006	0.123 ± 0.016	0.113 ± 0.010
Cystine	21:30	21:30	23:30	13:30	1.057 ± 0.006	1.043 ± 0.006	0.907 ± 0.035	0.941 ± 0.014	0.149 ± 0.040	0.103 ± 0.017
D-Glucosamine-6-Sulfate	9:30	11:30	11:30	21:30	1.052 ± 0.005	1.056 ± 0.009	0.952 ± 0.004	0.955 ± 0.004	0.100 ± 0.007	0.101 ± 0.013
Isoleucine	9:30	21:30	13:30	3:30	1.029 ± 0.004	1.034 ± 0.007	0.977 ± 0.002	0.982 ± 0.001	0.051 ± 0.005	0.052 ± 0.007
L-Glutamic Acid	21:30	21:30	13:30	13:30	1.038 ± 0.005	1.038 ± 0.008	0.970 ± 0.003	0.977 ± 0.002	0.069 ± 0.007	0.061 ± 0.01
L-Glutamine	9:30	7:30	9:30	1:30	1.030 ± 0.003	1.028 ± 0.003	0.968 ± 0.004	0.978 ± 0.002	0.063 ± 0.005	0.050 ± 0.005
Lysine	21:30	21:30	11:30	3:30	1.032 ± 0.004	1.044 ± 0.01	0.969 ± 0.004	0.975 ± 0.002	0.063 ± 0.007	0.068 ± 0.009
Methionine	21:30	21:30	1:30	11:30	1.035 ± 0.005	1.037 ± 0.008	0.970 ± 0.002	0.973 ± 0.003	0.065 ± 0.005	0.064 ± 0.007
O-Acetyl-L-Serine	21:30	21:30	13:30	13:30	1.038 ± 0.005	1.038 ± 0.008	0.970 ± 0.003	0.977 ± 0.002	0.069 ± 0.007	0.061 ± 0.01
Ornithine	21:30	21:30	13:30	15:30	1.039 ± 0.005	1.044 ± 0.01	0.972 ± 0.002	0.976 ± 0.003	0.067 ± 0.005	0.068 ± 0.01
Phenylalanine	21:30	21:30	1:30	1:30	1.033 ± 0.003	1.036 ± 0.008	0.977 ± 0.001	0.98 ± 0.002	0.056 ± 0.004	0.055 ± 0.008
Proline	15:30	21:30	7:30	5:30	1.046 ± 0.005	1.045 ± 0.005	0.942 ± 0.024	0.965 ± 0.004	0.104 ± 0.027	0.081 ± 0.007
Taurine	21:30	21:30	7:30	13:30	1.045 ± 0.005	1.052 ± 0.012	0.956 ± 0.004	0.950 ± 0.006	0.089 ± 0.006	0.102 ± 0.015
Threonine	13:30	21:30	13:30	9:30	1.039 ± 0.004	1.039 ± 0.008	0.975 ± 0.002	0.979 ± 0.002	0.062 ± 0.004	0.06 ± 0.009
Trans-4-Hydroxyproline	9:30	21:30	9:30	11:30	1.046 ± 0.007	1.043 ± 0.01	0.956 ± 0.007	0.961 ± 0.004	0.090 ± 0.011	0.083 ± 0.011
Tryptophan	19:30	21:30	1:30	5:30	1.025 ± 0.003	1.032 ± 0.007	0.981 ± 0.002	0.982 ± 0.002	0.043 ± 0.005	0.050 ± 0.008
Tyrosine	9:30	21:30	13:30	3:30	1.03 ± 0.004	1.032 ± 0.007	0.978 ± 0.002	0.985 ± 0.001	0.052 ± 0.006	0.048 ± 0.007
Valine	21:30	21:30	13:30	13:30	1.032 ± 0.004	1.044 ± 0.009	0.965 ± 0.006	0.960 ± 0.006	0.067 ± 0.009	0.084 ± 0.013
Hippuric Acid[B]	9:30	9:30	1:30	23:30	1.048 ± 0.007	1.051 ± 0.005	0.953 ± 0.007	0.951 ± 0.007	0.095 ± 0.013	0.100 ± 0.01
2,3-Dihydroxybenzoate	7:30	21:30	9:30	17:30	1.055 ± 0.004	1.063 ± 0.009	0.942 ± 0.008	0.937 ± 0.005	0.112 ± 0.008	0.126 ± 0.012
2-Quinoline-Carboxylic Acid	9:30	5:30	23:30	15:30	1.076 ± 0.01	1.066 ± 0.015	0.91 ± 0.017	0.912 ± 0.01	0.165 ± 0.018	0.154 ± 0.022
3-Methoxy-4-Hydroxy Mandelate	9:30	5:30	23:30	13:30	1.037 ± 0.006	1.035 ± 0.007	0.958 ± 0.008	0.95 ± 0.015	0.08 ± 0.013	0.085 ± 0.019
4-Hydroxy Phenylacetate-Mandelic Acid	13:30	21:30	21:30	21:30	1.053 ± 0.004	1.049 ± 0.006	0.948 ± 0.005	0.948 ± 0.007	0.105 ± 0.007	0.101 ± 0.007
Dihydroxy Mandelic Acid	9:30	21:30	21:30	17:30	1.055 ± 0.009	1.056 ± 0.008	0.906 ± 0.019	0.914 ± 0.014	0.15 ± 0.026	0.143 ± 0.018
Ferulate	15:30	19:30	3:30	9:30	1.142 ± 0.018	1.163 ± 0.01	0.868 ± 0.012	0.874 ± 0.014	0.274 ± 0.027	0.289 ± 0.016
Trans-Cinnamate[B]	21:30	21:30	5:30	5:30	1.04 ± 0.004	1.041 ± 0.011	0.97 ± 0.005	0.968 ± 0.003	0.07 ± 0.008	0.072 ± 0.013
3-Hydroxy Benzaldehyde	9:30	21:30	5:30	21:30	1.041 ± 0.007	1.052 ± 0.005	0.972 ± 0.003	0.97 ± 0.002	0.069 ± 0.008	0.081 ± 0.006
p-Cresol sulfate[C]	9:30	21:30	21:30	5:30	1.038 ± 0.003	1.039 ± 0.008	0.966 ± 0.005	0.966 ± 0.01	0.072 ± 0.007	0.072 ± 0.014
3α,7α,12α-Trihydroxy-5β-Cholestan-26-al	15:30	13:30	3:30	21:30	1.172 ± 0.022	1.184 ± 0.018	0.787 ± 0.020	0.728 ± 0.029	0.385 ± 0.035	0.456 ± 0.039
Coprocholic acid	9:30	21:30	21:30	11:30	1.032 ± 0.004	1.035 ± 0.008	0.961 ± 0.007	0.976 ± 0.003	0.071 ± 0.009	0.059 ± 0.01
27-Deoxy-5β-Cyprinol	21:30	5:30	11:30	21:30	1.074 ± 0.015	1.058 ± 0.012	0.956 ± 0.005	0.954 ± 0.007	0.118 ± 0.016	0.104 ± 0.017
3α,7α,24-Trihydroxy-	21:30	3:30	1:30	21:30	1.071 ± 0.006	1.078 ± 0.009	0.894 ± 0.012	0.900 ± 0.015	0.177 ± 0.015	0.178 ± 0.017

5β-Cholestanoyl-CoA										
7α-Hydroxy-3-oxo-4-Cholestenoate	9:30	21:30	9:30	21:30	1.028 ± 0.005	1.029 ± 0.007	0.976 ± 0.002	0.982 ± 0.002	0.052 ± 0.007	0.047 ± 0.008
Cholic Acid	21:30	21:30	5:30	23:30	1.081 ± 0.015	1.064 ± 0.007	0.948 ± 0.005	0.950 ± 0.003	0.133 ± 0.015	0.113 ± 0.008
Chenodeoxycholate	5:30	21:30	11:30	11:30	1.052 ±0.003	1.052 ± 0.008	0.950 ± 0.003	0.953 ± 0.004	0.102 ± 0.005	0.098 ± 0.011
Glycocholate	19:30	19:30	7:30	5:30	1.086 ± 0.010	1.073 ± 0.005	0.928 ± 0.004	0.902 ± 0.013	1.158 ± 0.011	0.170 ± 0.014
Chenodeoxy-glycocholate	15:30	21:30	1:30	23:30	1.048 ± 0.004	1.041 ± 0.006	0.955 ± 0.007	0.933 ± 0.012	0.093 ± 0.007	0.108 ± 0.016
Taurodeoxycholate	19:30	19:30	5:30	5:30	1.092 ± 0.009	1.083 ± 0.008	0.908 ± 0.008	0.899 ± 0.013	0.184 ± 0.014	0.183 ± 0.018
Lithocholic Acid[B]	15:30	23:30	21:30	21:30	1.050 ± 0.007	1.041 ± 0.005	0.945 ± 0.009	0.948 ± 0.009	0.105 ± 0.015	0.093 ± 0.012
Bilirubin	5:30	7:30	9:30	23:30	1.056 ± 0.01	1.046 ± 0.006	0.891 ± 0.032	0.926 ± 0.026	0.165 ± 0.039	0.12 ± 0.029
Biliverdin	5:30	7:30	19:30	23:30	1.047 ± 0.006	1.05 ± 0.006	0.924 ± 0.016	0.932 ± 0.017	0.123 ± 0.02	0.118 ± 0.021
10-Hydroxy decanoate	21:30	21:30	7:30	7:30	1.033 ± 0.003	1.039 ± 0.008	0.976 ± 0.003	0.974 ± 0.003	0.057 ± 0.004	0.065 ± 0.01
1-Oleoyl-rac-Glycerol	3:30	23:30	21:30	21:30	1.055 ± 0.009	1.05 ± 0.005	0.937 ± 0.008	0.937 ± 0.008	0.119 ± 0.014	0.112 ± 0.011
Arachidic Acid	5:30	3:30	21:30	11:30	1.063 ± 0.007	1.074 ± 0.007	0.932 ± 0.003	0.927 ± 0.004	0.132 ± 0.007	0.147 ± 0.01
Caprylic Acid	9:30	21:30	23:30	11:30	1.04 ± 0.006	1.047 ± 0.008	0.956 ± 0.006	0.959 ± 0.005	0.084 ± 0.01	0.088 ± 0.011
Docosahexaenoic Acid	21:30	23:30	17:30	21:30	1.043 ± 0.004	1.043 ± 0.006	0.949 ± 0.011	0.945 ± 0.013	0.094 ± 0.013	0.099 ± 0.017
Gamma Linolenic Acid	15:30	15:30	21:30	21:30	1.079 ± 0.012	1.077 ± 0.008	0.905 ± 0.012	0.913 ± 0.006	0.174 ± 0.014	0.164 ± 0.009
Heptadecanoate	1:30	3:30	17:30	21:30	1.051 ± 0.007	1.047 ± 0.007	0.951 ± 0.004	0.957 ± 0.004	0.100 ± 0.011	0.09 ± 0.01
Linoleate	15:30	23:30	21:30	21:30	1.047 ± 0.007	1.04 ± 0.004	0.942 ± 0.013	0.938 ± 0.012	0.106 ± 0.019	0.102 ± 0.015
Myristic Acid	15:30	13:30	7:30	19:30	1.068 ± 0.012	1.062 ± 0.011	0.933 ± 0.014	0.942 ± 0.008	0.136 ± 0.023	0.12 ± 0.017
Nonanoate	21:30	21:30	7:30	15:30	1.034 ± 0.003	1.048 ± 0.009	0.967 ± 0.006	0.962 ± 0.004	0.066 ± 0.008	0.086 ± 0.012
Palmitate	3:30	3:30	3:30	11:30	1.058 ± 0.008	1.046 ± 0.006	0.924 ± 0.024	0.936 ± 0.015	0.134 ± 0.024	0.111 ± 0.016
Palmitoleic Acid	7:30	1:30	21:30	5:30	1.069 ± 0.009	1.065 ± 0.007	0.901 ± 0.011	0.914 ± 0.007	0.168 ± 0.016	0.151 ± 0.013
Petroselinic Acid	3:30	1:30	21:30	19:30	1.061 ± 0.008	1.05 ± 0.008	0.914 ± 0.016	0.93 ± 0.012	0.146 ± 0.02	0.12 ± 0.018
Stearate	3:30	1:30	11:30	21:30	1.032 ± 0.003	1.03 ± 0.002	0.972 ± 0.003	0.968 ± 0.004	0.06 ± 0.004	0.062 ± 0.005
3-Hydroxy-3-Methyl Glutarate	21:30	21:30	3:30	5:30	1.064 ± 0.007	1.051 ± 0.008	0.945 ± 0.005	0.956 ± 0.005	0.12 ± 0.01	0.095 ± 0.012
α-Ketoglutaric Acid	21:30	23:30	1:30	19:30	1.099 ± 0.015	1.07 ± 0.008	0.919 ± 0.011	0.911 ± 0.011	0.182 ± 0.018	0.159 ± 0.017
α-Aminoadipate	21:30	21:30	23:30	11:30	1.046 ± 0.004	1.047 ± 0.007	0.967 ± 0.003	0.943 ± 0.017	0.08 ± 0.006	0.104 ± 0.018
Isatin (I.S.)[C]	18	21:30	11:30	9:30	1.053 ± 0.008	1.046 ± 0.006	0.964 ± 0.002	0.955 ± 0.005	0.090 ± 0.009	0.090 ± 0.008
Indole-3-Acetaldehyde[A]	9:30	21:30	15:30	13:30	1.032 ± 0.003	1.04 ± 0.005	0.969 ± 0.005	0.972 ± 0.005	0.064 ± 0.007	0.068 ± 0.009
Indole-3-Acetate[A]	21:30	21:30	19:30	15:30	1.034 ± 0.007	1.04 ± 0.009	0.971 ± 0.004	0.971 ± 0.002	0.063 ± 0.009	0.07 ± 0.011
Hypoxanthine[B]	21:30	15:30	13:30	5:30	1.059 ± 0.005	1.069 ± 0.008	0.963 ± 0.003	0.951 ± 0.005	0.095 ± 0.007	0.119 ± 0.009
Urate	21:30	11:30	17:30	13:30	1.055 ± 0.007	1.064 ± 0.009	0.961 ± 0.003	0.961 ± 0.004	0.095 ± 0.009	0.103 ± 0.011
Xanthine	21:30	21:30	15:30	13:30	1.041 ± 0.004	1.042 ± 0.01	0.967 ± 0.007	0.974 ± 0.002	0.073 ± 0.009	0.067 ± 0.011
Butyric Acid[A]	21:30	21:30	19:30	3:30	1.065 ± 0.005	1.058 ± 0.008	0.944 ± 0.006	0.948 ± 0.005	0.120 ± 0.010	0.110 ± 0.011
α-Hydroxy Isobutyric Acid	3:30	21:30	21:30	21:30	1.039 ± 0.006	1.045 ± 0.005	0.959 ± 0.003	0.966 ± 0.002	0.071 ± 0.008	0.078 ± 0.006
3-Methyl-2-oxo Valeric Acid	5:30	21:30	15:30	5:30	1.067 ± 0.006	1.059 ± 0.012	0.932 ± 0.004	0.953 ± 0.004	0.135 ± 0.007	0.107 ± 0.013
Cortisol	9:30	5:30	1:30	1:30	1.067 ± 0.005	1.064 ± 0.006	0.903 ± 0.008	0.909 ± 0.008	0.164 ± 0.01	0.154 ± 0.013
Cortisol 21-Acetate	21:30	23:30	23:30	1:30	1.084 ± 0.032	1.069 ± 0.011	0.930 ± 0.028	0.949 ± 0.004	0.153 ± 0.06	0.12 ± 0.014
Reichstein Substance	5:30	5:30	1:30	7:30	1.26 ± 0.027	1.298 ± 0.025	0.766 ± 0.012	0.768 ± 0.016	0.496 ± 0.027	0.53 ± 0.026
Acetylneuraminate	21:30	21:30	7:30	11:30	1.061 ± 0.006	1.062 ± 0.011	0.948 ± 0.005	0.945 ± 0.005	0.113 ± 0.01	0.107 ± 0.014
Arabinose	21:30	21:30	23:30	9:30	1.07 ± 0.005	1.074 ± 0.006	0.921 ± 0.012	0.907 ± 0.008	0.15 ± 0.014	0.167 ± 0.011
D-Ribose-5-Phosphate	19:30	19:30	1:30	3:30	1.114 ± 0.019	1.114 ± 0.015	0.861 ± 0.031	0.853 ± 0.037	0.253 ± 0.045	0.26 ± 0.05
Fructose/Mannose/Galactose	13:30	19:30	23:30	15:30	1.05 ± 0.009	1.047 ± 0.006	0.966 ± 0.004	0.976 ± 0.003	0.083 ± 0.012	0.071 ± 0.007
Galactarate	13:30	1:30	21:30	9:30	1.072 ± 0.008	1.069 ± 0.005	0.933 ± 0.005	0.92 ± 0.008	0.139 ± 0.011	0.149 ± 0.012
Gluconic Acid	13:30	21:30	3:30	21:30	1.054 ± 0.011	1.057 ± 0.009	0.948 ± 0.008	0.954 ± 0.006	0.105 ± 0.018	0.102 ± 0.014
Mannitol	13:30	21:30	3:30	3:30	1.062 ± 0.008	1.064 ± 0.006	0.874 ± 0.024	0.91 ± 0.013	0.188 ± 0.031	0.154 ± 0.017

S-Lactate-Glyceraldehyde	21:30	21:30	7:30	13:30	1.044 ± 0.004	1.044 ± 0.011	0.968 ± 0.002	0.969 ± 0.002	0.076 ± 0.004	0.075 ± 0.011
Indole-3-propionic acid[A]	23:30	3:30	1:30	23:30	1.046 ± 0.002	1.055 ± 0.008	0.970 ± 0.003	0.954 ± 0.004	0.076 ± 0.005	0.099 ± 0.011
Indoxyl sulfate (indican)[C]	19:30	21:30	21:30	5:30	1.039 ± 0.003	1.039 ± 0.009	0.955 ± 0.017	0.970 ± 0.004	0.083 ± 0.019	0.069 ± 0.011
Kynurenine[B]	19:30	21:30	11:30	9:30	1.043 ± 0.006	1.042 ± 0.005	0.957 ± 0.006	0.966 ± 0.003	0.086 ± 0.011	0.077 ± 0.006
N-methyl Serotonin (I.S.)[B]	9:30	21:30	17:30	21:30	1.070 ± 0.011	1.059 ± 0.009	0.940 ± 0.010	0.938 ± 0.014	0.129 ± 0.020	0.120 ± 0.020
Uridine	9:30	21:30	1:30	13:30	1.034 ± 0.005	1.036 ± 0.008	0.964 ± 0.005	0.958 ± 0.004	0.07 ± 0.008	0.078 ± 0.009
Retinoate	7:30	21:30	3:30	7:30	1.05 ± 0.005	1.056 ± 0.005	0.952 ± 0.004	0.933 ± 0.01	0.098 ± 0.006	0.122 ± 0.012
Creatinine[D]	19:30	1:30	3:30	9:30	1.054 ± 0.006	1.043 ± 0.006	0.940 ± 0.003	0.946 ± 0.005	0.115 ± 0.007	0.098 ± 0.009

[A]Derived solely from microbial metabolism. [B]Derived from both microbial and host metabolism. [C]Derived from host secondary metabolism of microbial metabolites. [D]Associated with shifts in gut microbes.

Table S5. Metabolite identification level

Metabolite type	Metabolite	Metabolite Identification Level
Acid	Oxalic Acid	5
Amino acids and derivatives	5-oxo-D-Proline	1
	Alanine	1
	Aspartate	1
	β-Alanine/Sarcosine	1
	Citrulline	1
	Cystine	1
	D-Glucosamine-6-Sulfate	1
	L-Isoleucine	1
	L-Glutamic Acid	1
	L-Glutamine	1
	L-Lysine	1
	L-Methionine	1
	O-Acetyl-L-Serine	1
	L-Ornithine	1
	L-Phenylalanine	1
	L-Proline	1
	Taurine	1
	L-Threonine	1
	Trans-4-Hydroxyproline	1
	Tryptophan	1
	L-Tyrosine	1
	L-Valine	1
Aryl acids	Hippuric acid	1
	2,3-Dihydroxybenzoate	5
	2-Quinoline-Carboxylic Acid	1
	3-Methoxy-4-Hydroxymandelate	5
	4-Hydroxyphenylacetate; Mandelic Acid	5
	Dihydroxymandelic Acid	5
	Ferulate	1
	Trans-Cinnamate	1
Aryl aldehyde	3-Hydroxybenzaldehyde	5
Aryl Sulfate	p-Cresol sulfate[C]	5
Bile acids primary precursors	3α,7α,12α-Trihydroxy-5β-Cholestan-26-al	5
	27-Deoxy-5β-Cyprinol	5
	3α,7α,24-Trihydroxy-5β-Cholestanoyl-CoA	5
	7α-Hydroxy-3-oxo-4-Cholestenoate	5
Bile acids primary	Cholic acid	5
	Chenodeoxycholate	1
Bile acids Glyco-conjugated	Glycocholate	1
	Chenodeoxyglycocholate	5
Bile acids Tauro-conjugated	Taurodeoxycholate	5
Bile acids secondary	Lithocholic acid[A]	5
	Coprocholic acid	5
	Bilirubin	1

Bile component	Biliverdin	1
Fatty acids	10-Hydroxydecanoate	1
	1-Oleoyl-rac-Glycerol	1
	Arachidic Acid	5
	Caprylic Acid	5
	Docosahexanoic Acid	5
	Gamma Linolenic Acid	1
	Heptadecanoate	5
	Linoleate	1
	Myristic Acid	5
	Nonanoate	5
	Palmitate/ Hexadexanoic acid	5
	Palmitoleic Acid	5
	Petroselinic Acid	5
	Stearate	1
Glutaric acid derivatives	3-Hydroxy-3-Methylglutarate	5
	α-Ketoglutaric Acid	5
Homoisocitrate derivative	α-Aminoadipate	1
Indoles and derivatives	Isatin (I.S.)	5
	Indole-3-Acetaldehyde	1
	Indole-3-Acetate	1
Purines and derivatives	Hypoxanthine	1
	Urate	1
	Xanthine	1
SCFA and derivatives	Butyric acid	5
	α-Hydroxyisobutyric Acid	5
Short chain keto acids and derivatives	3-Methyl-2-oxovaleric Acid	5
Steroids and derivatives	Cortisol	1
	Cortisol 21-Acetate	1
	Reichstein Substance	5
Sugars and derivatives	N-Acetylneuraminate	1
	Arabinose	5
	D-Ribose-5-Phosphate	1
	Fructose/Mannose/Galactose	1
	Galactarate	1
	D-Gluconic Acid	1
	Mannitol	1
	S-Lactate-Glyceraldehyde	5
Tryptophan catabolites	Indole-3-propionic acid	5
	Indoxyl sulfate	5
	Kynurenine	1
	N-methylserotonin (I.S.)	5
Uracil containing precursor and derivatives	Uridine	1
Vitamin	Retinoate	1
Other	Creatinine	1
